# Deep limit order book forecasting: a microstructural guide

**DOI:** 10.1080/14697688.2025.2522911

**Published:** 2025-07-22

**Authors:** Antonio Briola, Silvia Bartolucci, Tomaso Aste

**Affiliations:** † Department of Computer Science, University College London, London, WC1E 6EA, UK; ‡ Systemic Risk Centre, London School of Economics, London, WC2A 2AE, UK

**Keywords:** Econophysics, Market microstructure, Limit order book, High frequency trading, Deep learning, C32, C53, C55, G14

## Abstract

We exploit cutting-edge deep learning methodologies to explore the predictability of high-frequency Limit Order Book mid-price changes for a heterogeneous set of stocks traded on the NASDAQ exchange. In so doing, we release ‘LOBFrame’, an open-source code base to efficiently process large-scale Limit Order Book data and quantitatively assess state-of-the-art deep learning models' forecasting capabilities. Our results are twofold. We demonstrate that the stocks' microstructural characteristics influence the efficacy of deep learning methods and that their high forecasting power does not necessarily correspond to actionable trading signals. We argue that traditional machine learning metrics fail to adequately assess the quality of forecasts in the Limit Order Book context. As an alternative, we propose an innovative operational framework that evaluates predictions' practicality by focusing on the probability of accurately forecasting complete transactions. This work offers academics and practitioners an avenue to make informed and robust decisions on the application of deep learning techniques, their scope and limitations, effectively exploiting emergent statistical properties of the Limit Order Book.

## 1. Introduction

Financial markets operate as highly stochastic environments characterized by a low signal-to-noise ratio, where a diverse set of market participants interacts at varying time scales with asymmetric access to information and differing trading capabilities (Bouchaud *et al.* 2009, Farmer and Skouras 2013, Scholl *et al.* 2021). Managing the complexity of these interactions requires modern exchanges to rely on sophisticated computerized systems that constantly collect, process, and organize the continuous flux of orders. These systems facilitate order matching while ensuring transaction fairness. A key component of such systems is the Limit Order Book (LOB), which provides real-time access to market supply and demand through a structured queue of buy and sell orders. Execution priority in most modern exchanges follows a first-in, first-out (FIFO) mechanism (Bouchaud *et al.* 2018), influencing the price formation process and market dynamics (Abergel *et al.* 2016, O'hara 2018).

A crucial aspect of modern trading is High-Frequency Trading (HFT), a strategy that gain an edge through speed, allowing certain traders to act on information not yet accessible to others (Lehalle and Laruelle 2018). Unlike traditional trading approaches, HFT does not rely on fundamental valuation but instead exploits market microstructure patterns, often generating noise and reinforcing price unpredictability (Bouchaud *et al.* 2009). While some studies suggest that HFT enhances market efficiency, others argue it may exacerbate instability (Markets 2009, Zhang 2010, Zhang and Powell 2011, Cartea and Penalva 2012, Jarrow and Protter 2012). The role of HFT in shaping LOB dynamics underscores the importance of accurate short-term price forecasting models, which can aid in understanding market behavior and improving trading strategies.

Recent advances in deep learning have significantly transformed the landscape of LOB price forecasting, introducing models capable of capturing complex, non-linear relationships within high-frequency data (Dixon 2018, Sirignano 2019, Zhang *et al.* 2019, Briola *et al.* 2020). However, despite the growing interest in applying deep learning techniques to LOB forecasting, significant challenges remain. Many academic studies focus on algorithmic improvements, while neglecting the practical applicability of their findings. Moreover, the field suffers from a lack of standardized evaluation methodologies and open-source tools to facilitate model benchmarking and replication.

To bridge this gap, we introduce ‘LOBFrame’,^1^ an open-source framework designed to standardize the preprocessing, modeling, and evaluation of deep learning models for LOB forecasting. LOBFrame addresses three fundamental challenges in the field:

- **Microstructural Understanding**: By linking stocks' ‘predictability rate’ (i.e. the probability of correctly forecasting the direction of mid-price movements over a certain time horizon) directly to their microstructural properties, such as tick size and liquidity, LOBFrame provides a framework for evaluating model effectiveness beyond standard classification metrics.
- **Reproducibility and Accessibility**: Unlike prior work that often relies on proprietary datasets and non-reproducible methodologies, LOBFrame offers an open-source, modular codebase, which eases the integration with new forecasting models.
- **Practical Benchmarking**: Our framework enables rigorous model evaluation, bridging the gap between theoretical forecasting performance and real-world usability by incorporating simulation-to-reality gap analysis (Prata *et al.* 2023). In particular, we introduce a new metric, i.e. the probability $p_{T}$ to execute a correct transaction, to evaluate models' performance beyond standard machine learning metrics.

In this study, we focus on the interplay between microstructural characteristics and LOB forecasting accuracy. Using a diverse set of 15 NASDAQ stocks, we assess the predictability of mid-price movements across varying time horizons and evaluate the performance of a state-of-the-art deep learning model, DeepLOB (Zhang *et al.* 2019). While DeepLOB has demonstrated strong performance in prior studies, our analysis explores why forecasts succeed or fail in relation to underlying LOB properties. Furthermore, while this paper uses the state-of-the-art deep learning model DeepLOB as a case study, LOBFrame is designed to be model-agnostic. Our approach allows for seamless integration of alternative forecasting models, including traditional machine learning algorithms, and other deep learning architectures. The broader goal of this work is to establish a benchmark methodology for assessing LOB forecasting models, encouraging further advancements in the field through an open, reproducible, and interpretable approach.

The rest of the paper is structured as follows: section 2 provides an overview of LOB mechanics, followed by a discussion of related literature in section 3. In section 4, we describe the dataset used in our experiments, while section 5 introduces the LOBFrame framework and experimental setup. Section 6 explores the microstructural characteristics of the selected stocks, and section 7 presents our forecasting results and their implications. Finally, section 8 concludes the paper providing a unified view of market microstructure-informed deep learning methods for LOB forecasting, with an overview on the open challenges in the field.

## 2. Limit order book

**Figure 1.**
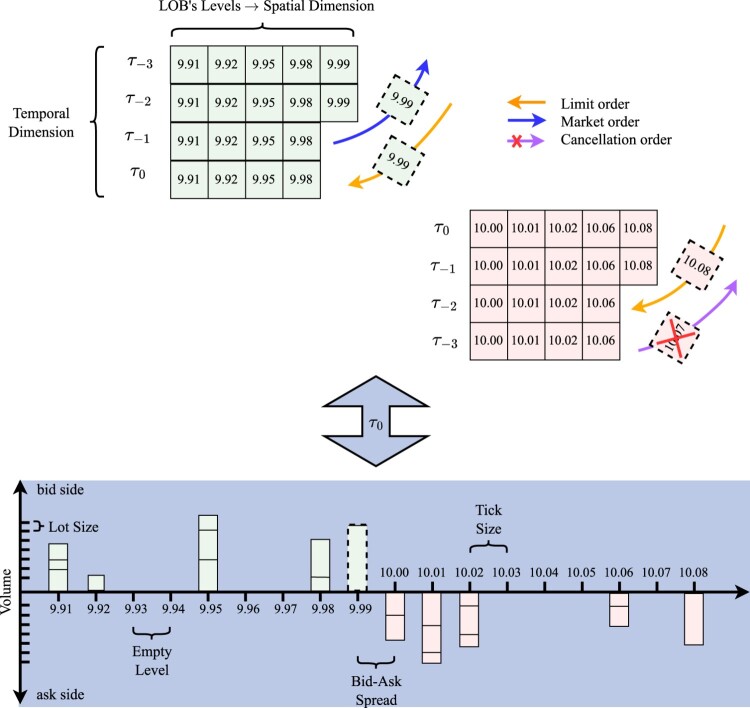
Pictorial representation of the LOB. In the upper part of the Figure, we show the dynamical evolution of the LOB price levels as a consequence of the submission of limit, market and cancellation orders; in the lower part of the Figure, we show a static view of a LOB snapshot (i.e. $L(\tau_{0})$) including also the volumes.

The majority of modern exchanges utilize an electronic system that stores and matches agents' trading intentions. This system operates on a data structure known as the ‘Limit Order Book’ (LOB) (see figure 1). Each security has its own LOB, which gives traders simultaneous access to the currently visible market's supply and demand. In this context, the price formation of an arbitrary security is a self-organized process driven by the submission and cancellation of orders (Briola *et al.* 2021).

An order can be considered a visible declaration of a market participant's intention to buy or sell a fixed amount of an asset's shares at a specified price. Its execution is subordinated to finding a counterpart willing to trade at the same conditions. Following the notation proposed by Bouchaud *et al.* (2018), an order o is formally defined as a tuple $\left(\epsilon_{o},p_{o},v_{o},\tau_{o}\right)$ where:

- $\epsilon_{o}$ indicates the sign or direction at which a given asset is traded. Conventionally, buy (or bid) orders have a positive sign ϵ=+1, while sell (or ask) orders have a negative sign ϵ=−1.
- $p_{o}$ indicates the price a trader wants to trade a given asset. Orders can be submitted at prices belonging to a discrete set, constituting the LOB's price levels (or quotes). The smallest price increment is known as ‘tick size’ (*θ*), which, on the NASDAQ exchange, is fixed and equal to $0.01 for all the securities.^2^
- $v_{o}$ indicates the number of asset shares a trader wants to exchange. Orders can be submitted on a discrete set of volumes, constituting LOB's volume levels. The smallest volume increment, which determines the minimum distance between two consecutive volume levels, is known as ‘lot size’, and, on the NASDAQ exchange, it is fixed and equal to 1 for all the securities.
- $\tau_{o}$ indicates the time an order is submitted, and it is a continuous variable (typically known with a precision of up to the nanoseconds).

There are three main families of orders that can be submitted: (i) limit orders; (ii) market orders; (iii) cancellation orders. A limit order represents an intention to buy or sell a fixed amount of an asset at a price different from the current best available matching price on the opposite side of the LOB. There is no guarantee of execution for this type of orders. A limit order is typically subject to lower transaction costs (i.e. the costs of transferring ownership rights (Niehans 2018)) since it actively provides liquidity to the LOB. A market order represents an intention to buy or sell a fixed amount of shares at the current best available matching price. If its volume is higher than the one supporting the best quote on the opposite side of the LOB, the remaining amount is executed against active orders at deeper price levels (sitting further away from the best quote). A market order is typically subject to higher transaction costs since it reduces the liquidity available in the LOB. A cancellation order represents an intention to fully or partially delete an active limit order. It is typically not subject to any transaction cost. The majority of executed trades often result from aggressive limit orders, rather than solely from market orders (Bouchaud *et al.* 2018).

All the elements introduced above allow us to define the LOB as a collection of unmatched (active) limit orders for a given asset, on a given platform, at time *τ* (Briola *et al.* 2021) (see figure 1). We represent it as a multivariate time-series L, where each $L(\tau )\in R^{4L}$ is a LOB record characterized by *L* price/volume levels (Prata *et al.* 2023). More specifically, L(τ) can be written in the form of $\{P^{s}(\tau ),V^{s}(\tau ){\}}_{s\in \{\mathrm{ask},\mathrm{bid}\}}$, where $P^{\mathrm{ask}}(\tau ),P^{\mathrm{bid}}(\tau )\in R^{L}$ are the sets of prices on the ask and bid side, and $V^{\mathrm{ask}}(\tau ),V^{\mathrm{bid}}(\tau )\in R^{L}$ are the sets of volumes on the ask and bid side, respectively:

(1) $$L(\tau )=\{p_{\ell }^{\mathrm{ask}}(\tau ),v_{\ell }^{\mathrm{ask}}(\tau ),p_{\ell }^{\mathrm{bid}}(\tau ),v_{\ell }^{\mathrm{bid}}(\tau ){\}}_{\ell =1}^{L}.$$

This means that, ∀ τ∈{1,…,N} and ∀ ℓ∈{1,…,L} on the *s* side, $v_{\ell }^{s}(\tau )$ shares can be sold or bought at price $p_{\ell }^{s}(\tau )$. The mid-price $m_{\tau }$ of the stock at time *τ*, is defined as the average between the best ask price (i.e. $p_{1}^{\mathrm{ask}}(\tau )$) and the best bid price (i.e. $p_{1}^{\mathrm{bid}}(\tau )$), $m_{\tau }=\frac{p_{1}^{\mathrm{ask}}(\tau )+p_{1}^{\mathrm{bid}}(\tau )}{2}$. The bid-ask spread $\sigma_{\tau }$ of the stock at time *τ*, is defined as the difference between the best ask price and the best bid price, $\sigma_{\tau }=p_{1}^{\mathrm{ask}}(\tau )-p_{1}^{\mathrm{bid}}(\tau )$.

## 3. Related work

Market microstructure and automated learning modeling of LOB dynamics are two continuously evolving research areas. In this Section, we provide a subset of core references to works that allow the reader to navigate the broader universe of related literature.

Market microstructure entails the analysis of how traders' intentions are translated into prices and volumes (Madhavan 2000, Biais *et al.* 2005). The aim is to understand fundamental issues and phenomena, such as characterizing price formation mechanisms (Mike and Farmer 2005, Bonart and Lillo 2018, Lillo 2021) and quantifying the impact of asymmetric information (Glosten and Milgrom 1985, Kyle 1985). In terms of markets' dynamics, price jumps (Tóth *et al.* 2009, Zheng *et al.* 2012, Cont and De Larrard 2013, Marcaccioli *et al.* 2022) and flash crashes events (Brewer *et al.* 2013, Kirilenko *et al.* 2017, Paddrik *et al.* 2017, Turiel and Aste 2021, 2022) have been extensively studied. Modeling and analyzing transaction costs (e.g. price impact (Avellaneda and Stoikov 2008, Eisler *et al.* 2012, Cont *et al.* 2014, Mastromatteo *et al.* 2014)), and optimal order execution is also among core areas of investigation, especially in the context of HFT (Hollifield *et al.* 2004, Cartea and Jaimungal 2015, Lehalle and Mounjid 2017, Cartea *et al.* 2018). In this paper, we analyze a set of microstructural properties that can be used to characterize and classify stocks. For a complete review and deep discussion of the emergent statistical properties of stocks, we refer the reader to the comprehensive book by Bouchaud *et al.* (2018). Specifically, we focus on spread and liquidity (e.g. depth at best), whose behaviors have been extensively studied for different types of stocks in various markets, evidencing typical intra-day behaviors and statistical properties (Chakraborti *et al.* 2011, Abergel *et al.* 2016, Lehalle and Mounjid 2017). In our analysis, we also reference a more recently introduced measure, namely the ‘information richness’ (IR) (Kolm *et al.* 2023), which characterizes the stocks' activity in terms of the number of events occurring at the best levels of the LOB.

In this paper, we directly link the microstructural assessment of the LOB properties with mid-price changes forecasting performance of a state-of-the-art deep learning model, namely the DeepLOB (Zhang *et al.* 2019), specifically crafted to handle such data. Regarding the automated learning modeling of the LOB, it is useful to organize the related literature into three main areas of interest: (i) the study of linear models and regression analysis tools for LOB features extraction (Alvim *et al.* 2010, Zheng *et al.* 2012, Cenesizoglu *et al.* 2014, Detollenaere and D'hondt 2017, Panayi *et al.* 2018); (ii) the study of non-linear deep learning models for short-term price forecasting (Kearns and Nevmyvaka 2013, Passalis *et al.* 2017, Tsantekidis *et al.* 2017b, Tran *et al.* 2018, Nousi *et al.* 2019, Zhang *et al.* 2019, Briola *et al.* 2020, Passalis *et al.* 2020, Tsantekidis *et al.* 2020, Tran *et al.* 2021, Zhang and Zohren 2021, Guo and Chen 2023); (iii) the study of reinforcement learning methods for automated trading (Nevmyvaka *et al.* 2006, Zarkias *et al.* 2019, Kumar 2020, Briola *et al.* 2021, Gašperov and Kostanjčar 2021, Gašperov *et al.* 2021, Tsantekidis *et al.* 2021, Kumar 2023, Frey *et al.* 2023, Nagy *et al.* 2023, Tsantekidis *et al.* 2023). Linear models are easy to estimate and capture in a simple way the trends, linear correlations and autocorrelations in the state variables. Even if largely explored in the past years, their limitations have been recently analytically characterized in the work by Sirignano and Cont (2021), where the authors, supported by an abundant empirical and econometric literature documenting nonlinear effects in financial time series, demonstrate the necessity of flexible deep learning-based models to capture nonlinear relations between state variables and price moves in LOBs. For this reason, in the current paper, we direct our attention toward the second macro-area listed above. Assessing non-linear deep learning models for short-term price forecasting, we underline 3 main issues that are common to the majority of referenced research works: (i) the usage of only one simplistic dataset, namely the FI-2010 dataset (Ntakaris *et al.* 2018) as benchmark dataset; (ii) the lack of data analysis for proprietary LOB data; (iii) the difficulty in experiments' reproducibility. FI-2010 consists of 10 trading days LOB data from 5 Finnish companies traded on the NASDAQ Nordic stock market. It records 4M events sampled at intervals of 10 LOB updates, resulting in ≈395K events. This dataset represents the first and unique experiment to provide a standard benchmark for research in LOB forecasting. Even if remarkable, the outcome of this attempt presents some significant limitations. The dataset comes in an already pre-processed (filtered, normalized, and labeled) format so that the original LOB cannot be backtracked, thus hampering thorough experimentation. In addition to this, the dataset is too simplistic, leaving ample space for models' overfitting (Prata *et al.* 2023), consequently undermining their robustness when tested in real-world scenarios. Using this dataset as a benchmark for deep learning models represents the first cause of the so-called ‘simulation-to-reality’ gap (Liu *et al.* 2022, Zaznov *et al.* 2022). The singular characteristics of this benchmark dataset lead us to discuss the second type of criticality. Proprietary LOB data are considered sensible data, owned and managed by private financial institutions (Briola and Aste 2022, Vidal-Tomás *et al.* 2023, Briola *et al.* 2023a) with few third-party vendors, who only distribute exchange-specific historical samples. This makes academic research in the field highly dependent on data sources and the generalization capabilities of developed models questionable. Moreover, an accurate description and quantitative analysis of the dataset are often lacking, making comparisons of models' performances on stocks traded on different exchanges even more unreliable, thus representing a barrier towards experiments' reproducibility (Prata *et al.* 2023). Similarly, the code used to conduct the analysis is also rarely shared, directly hampering a meaningful comparison between different approaches.

In the broader context of the questions addressed in this paper, closely related works are those by Lucchese *et al.* (2022), Prata *et al.* (2023), Kolm *et al.* (2023) and Aït-Sahalia *et al.* (2022). The common aspect that links all these research papers is a significant effort in investigating the reasons why deep learning models are effective only in specific scenarios. In the work by Lucchese *et al.* (2022), the authors isolate some important factors that guarantee a successful forecast, including working with what they define ‘high-frequency stocks’, L2 data (i.e. all available bid and ask prices and corresponding volumes) and an order-flow representation of the LOB. In their narrative, the authors are particularly interested in statistically assessing the performance's degradation at longer prediction horizons. The work by Prata *et al.* (2023) anticipates some of the technical drawbacks discussed in this Section and highlights the influence of volatility clusters on forecasting models' performances. In the work by Kolm *et al.* (2023), the authors introduce the concept of ‘information-rich stocks’ and show how automated learning models can handle them more easily. Lastly, in the work by Aït-Sahalia *et al.* (2022), the authors succeed in isolating some of the variables that are thought to be among the more responsible for driving stocks' predictability.

## 4. Data

In this work we consider 15 stocks from different sectors and industries, all traded on the NASDAQ exchange (NASDAQ 2023a). For each of them, we use high quality, tick-by-tick, LOB data from the LOBSTER provider (LOBSTER 2023). To determine stocks' sector and industry affiliation, we follow the taxonomy proposed by the NASDAQ exchange itself (NASDAQ 2023b); in this context, the strong heterogeneity of our choices ensures robustness to results. As one can see from table 1, we consider 5 stocks belonging to the ‘Technology’ sector (i.e. AAPL, GOOG, IBM, NVDA, ORCL), 3 stocks belonging to the ‘Health Care’ sector (i.e. ABBV, PFE, PM), 3 stocks belonging to the ‘Telecommunications’ sector (i.e. CHTR, CSCO, VZ), 2 stocks belonging to the ‘Finance’ sector (i.e. BAC, GS), 1 stock belonging to the ‘Consumer Staples’ sector (i.e. KO) and 1 stock belonging to the ‘Consumer Discretionary’ sector (i.e. MCD). We consider the entire trading period from 2017 to 2019, ensuring to treat only stocks maintaining a large- (i.e. 10B-200B) to mega- (i.e. ≥200B) capitalization.

**Table 1.** Overview of the stocks used in the papers. For each asset we report the ticker, the extended name, the sector, the industry and the capitalization during 2017, 2018 and 2019. To determine stocks' sector and industry affiliation, we follow the taxonomy proposed by the NASDAQ exchange itself (NASDAQ 2023b). To determine stock's capitalization we rely on the data provided by CompaniesMarketCap.com (Companies Market Cap 2024). For each year, we report the average capitalization, standard deviation, as well as the 35th and 75th percentiles.

Ticker	Stock name	Sector	Industry	Capitalization (2017)	Capitalization (2018)	Capitalization (2019)
AAPL	Apple, Inc.	Technology	Computer Manufacturing	$860.88 B	$746.07 B	$1.287 T
ABBV	AbbVie, Inc.	Health Care	Biotechnology: Pharmaceutical Preparations	$154.39 B	$136.33 B	$130.94 B
BAC	Bank of America Corporation	Finance	Major Banks	$307.91 B	$238.25 B	$311.20 B
CHTR	Charter Communications, Inc.	Telecommunications	Cable & Other Pay Television Services	$83.94 B	$64.21 B	$101.85 B
CSCO	Cisco Systems, Inc.	Telecommunications	Computer Communications Equipment	$189.34 B	$194.81 B	$203.45 B
GOOG	Alphabet, Inc.	Technology	Computer Software: Programming, Data Processing	$729.45 B	$723.55 B	$921.13 B
GS	Goldman Sachs Group, Inc.	Finance	Investment Bankers/Brokers/Service	$96.09 B	$61.43 B	$79.86 B
IBM	International Business Machines Corporation	Technology	Computer Manufacturing	$142.03 B	$101.44 B	$118.90 B
KO	Coca-Cola Company	Consumer Staples	Beverages (Production/Distribution)	$195.47 B	$202.08 B	$236.89 B
MCD	McDonald's Corporation	Consumer Discretionary	Restaurants	$137.21 B	$136.21 B	$147.47 B
NVDA	NVIDIA Corporation	Technology	Semiconductors	$117.26 B	$81.43 B	$144.00 B
ORCL	Oracle Corporation	Technology	Computer Software: Prepackaged Software	$195.72 B	$162.03 B	$169.94 B
PFE	Pfizer, Inc.	Health Care	Biotechnology: Pharmaceutical Preparations	$215.89 B	$249.54 B	$216.82 B
PM	Philip Morris International, Inc.	Health Care	Medicinal Chemicals and Botanical Products	$164.09 B	$103.78 B	$132.39 B
VZ	Verizon Communications, Inc.	Telecommunications	Telecommunications Equipment	$215.92 B	$232.30 B	$253.93 B
Mean				$253.71 B	$228.90 B	$297.05 B
Standard deviation				$220.29 B	$207.36 B	$329.04 B
35th Percentile				$153.15 B	$132.97 B	$142.84 B
75th Percentile				$ 215.90 B	$ 235.28 B	$245.41 B

To train our model, we use only a portion of the entire dataset. For each year, we choose 45 consecutive days of training, 5 days of validation and 10 consecutive days of testing (see table 2). It is worth noting that the 5 days of the validation set are not consecutive and are randomly chosen from the same period of the training set. This choice guarantees greater robustness in the validation step, and it is made possible by the adopted standardization procedure, which prevents any data leakage. In line with what is suggested by Lucchese *et al.* (2022), a 5-days feature-wise rolling window *z*-score normalization is applied to the data. This procedure differs from the others used in most of the related literature (which usually standardizes the entire training dataset at once based on the overall statistics) (Zhang *et al.* 2019) and guarantees greater effectiveness in an evolving and strongly non-stationary environment like the LOB. All the experiments presented in the current work are conducted using the first *L* = 10, non-empty LOB levels (see equation (1)). Data (LOBSTER 2023) are originally made of two separate files: (i) the ‘message file’ lists every market-, limit- and cancellation order, reporting the arrival time, event type, id, size, price and direction; (ii) the ‘orderbook file’ describes the market state (i.e. the total volume of buy or sell orders at each price level) immediately after an event occurs. These files are jointly processed as described by Lucchese *et al.* (2022) by (i) removing crossed quotes; (ii) collapsing states occurring at the same timestamp (to nanoseconds precision) to the last state; and (iii) removing the effects of potential auction calls by considering only events happening between 9:40 am (Eastern Time) and 03:50 pm (Eastern Time). This last choice is made following the suggestion by Briola *et al.* (2021) to exclude from experiments the first and the last 10 minutes of each trading day due to the widely different dynamics and higher volatility that usually affect the market's opening and closing periods. The reader should be aware that trading does not occur on weekends or public holidays, so these days are excluded from all the analyses.

**Table 2.** Basic structure of the datasets used during the training, validation and test stages. For each year, for the training and test set, we report the starting and the ending day (which are included), while, for the validation set, we report all the days in an extended way. It is worth noticing that weekends and public holidays are not trading days and, consequently, do not belong to any of the datasets.

	Training		Validation	Test	
Year	From	To	Days	From	To
			Mar 23, Apr 05,		
2017	Mar 13	May 22	Apr 13, Apr 18,	May 23	Jun 06
			May 02		
			Aug 15, Aug 16,		
2018	Aug 09	Oct 18	Sep 19, Sep 26	Oct 19	Nov 01
			Oct 03		
			Jun 14, Jun 27,		
2019	Jun 04	Aug 13	Jul 08, Jul 10,	Oct 19	Nov 01
			Jul 24		

In this work, based on the microstructural properties of a given stock, we are interested in studying the predictability of the direction of mid-price changes at different time horizons when such a movement is larger than or equal to *θ*. For the sake of readability, we will refer to these mid-price differences as ‘increments’, stressing that we refrain from using relative returns nor logarithmic ones. We decide to use the simple difference in mid-prices to gain higher control over the amplitude of the change at different time horizons, preserving, at the same time, the stationarity property of the resulting time-series. Many alternatives have been proposed as target variables in the literature (Tsantekidis *et al.* 2017a, Ntakaris *et al.* 2018, Zhang *et al.* 2019, Lucchese *et al.* 2022). All of them are based on the usage of the log-return as a fundamental quantity and apply different smoothing methods to prevent a strong fit between labels and actual prices. Even if acceptable from an academic perspective, the practicability of these choices is unclear since they are designed to characterize mid-price trends (not immediate changes), leaving a reduced control over tick-by-tick changes, which are of higher interest in the development of high-frequency trading strategies.

In this paper, we consider 3 different horizons HΔτ∈{10,50,100} and, for each of them, the labeling step can be described as follows:

(2) $$\left\{ \begin{array}{l} (m_{\tau +\Delta \tau }-m_{\tau })\leq -\theta \to -1\to \mathrm{Down},\\ -\theta <(m_{\tau +\Delta \tau }-m_{\tau })<+\theta \to 0\to \mathrm{Stable},\\ (m_{\tau +\Delta \tau }-m_{\tau })\geq +\theta \to 1\to \mathrm{Up}, \end{array}\right.$$

where *θ* is the tick size and $m_{\tau }$ is the mid-price at time *τ*.^3^ It is worth noting that horizons are always defined in terms of LOB updates (which are unevenly spaced), while physical time is never used. Tables 3, 4 and 5 report the stocks' average daily class distribution for the training, validation and test set, computed across the 3-year analysis period, for HΔτ∈{10,50,100}. Generally speaking, it is always possible to detect imbalances. Their evolution across horizons, however, varies for different groups (or sets) of stocks (notice that, in tables 3, 4 and 5, groups are separated by horizontal lines). The groups' separation into so-called small-tick stocks (group 1), medium-tick stocks (group 2) and large-tick stocks (group 3) will be formally described in section 6 in relation to the microstructural properties displayed by the financial assets.

**Table 3.** Stocks' average daily class distribution for the training set, computed across the 3-year analysis period, for HΔτ∈{10,50,100}.

	H10			H50			H100		
Ticker	Down	Stable	Up	Down	Stable	Up	Down	Stable	Up
CHTR	2.19e+04	1.93e+04	2.11e+04	2.95e+04	4.67e+03	2.81e+04	3.05e+04	2.24e+03	2.96e+04
GOOG	8.82e+04	1.92e+05	8.66e+04	1.47e+05	7.31e+04	1.47e+05	1.64e+05	3.71e+04	1.66e+05
GS	3.96e+04	4.18e+04	3.96e+04	5.50e+04	1.12e+04	5.49e+04	5.77e+04	6.12e+03	5.72e+04
IBM	4.10e+04	7.29e+04	4.13e+04	6.56e+04	2.40e+04	6.56e+04	7.04e+04	1.46e+04	7.03e+04
MCD	3.46e+04	5.60e+04	3.50e+04	5.32e+04	1.84e+04	5.39e+04	5.69e+04	1.10e+04	5.77e+04
NVDA	1.18e+05	1.27e+05	1.18e+05	1.62e+05	3.80e+04	1.63e+05	1.69e+05	2.42e+04	1.70e+05
AAPL	2.06e+05	4.59e+05	2.06e+05	3.36e+05	1.98e+05	3.37e+05	3.67e+05	1.33e+05	3.70e+05
ABBV	4.00e+04	1.07e+05	3.98e+04	6.95e+04	4.82e+04	6.90e+04	7.77e+04	3.17e+04	7.73e+04
PM	3.68e+04	9.05e+04	3.69e+04	6.37e+04	3.63e+04	6.42e+04	7.02e+04	2.30e+04	7.09e+04
BAC	1.24e+04	4.59e+05	1.23e+04	4.32e+04	3.98e+05	4.30e+04	6.91e+04	3.46e+05	6.87e+04
CSCO	2.36e+04	4.51e+05	2.39e+04	7.32e+04	3.52e+05	7.33e+04	1.12e+05	2.75e+05	1.12e+05
KO	1.44e+04	2.14e+05	1.44e+04	4.17e+04	1.59e+05	4.15e+04	6.03e+04	1.22e+05	6.00e+04
ORCL	2.63e+04	3.15e+05	2.62e+04	6.93e+04	2.29e+05	6.93e+04	9.75e+04	1.73e+05	9.75e+04
PFE	1.85e+04	2.97e+05	1.85e+04	5.25e+04	2.29e+05	5.25e+04	7.65e+04	1.82e+05	7.62e+04
VZ	2.45e+04	2.62e+05	2.44e+04	6.52e+04	1.81e+05	6.49e+04	8.97e+04	1.33e+05	8.91e+04

**Table 4.** Stocks' average daily class distribution for the validation set, computed across the 3-year analysis period, for HΔτ∈{10,50,100}.

	H10			H50			H100		
Ticker	Down	Stable	Up	Down	Stable	Up	Down	Stable	Up
CHTR	1.90e+04	1.82e+04	1.87e+04	2.66e+04	3.95e+03	2.54e+04	2.76e+04	1.71e+03	2.66e+04
GOOG	6.73e+04	1.56e+05	6.70e+04	1.15e+05	6.13e+04	1.15e+05	1.30e+05	3.16e+04	1.29e+05
GS	4.10e+04	4.77e+04	4.10e+04	5.83e+04	1.38e+04	5.76e+04	6.12e+04	7.58e+03	6.10e+04
IBM	3.59e+04	6.92e+04	3.58e+04	5.86e+04	2.34e+04	5.90e+04	6.31e+04	1.43e+04	6.35e+04
MCD	3.19e+04	5.73e+04	3.24e+04	5.03e+04	1.92e+04	5.21e+04	5.41e+04	1.12e+04	5.63e+04
NVDA	1.33e+05	1.37e+05	1.32e+05	1.80e+05	4.29e+04	1.79e+05	1.87e+05	2.77e+04	1.87e+05
AAPL	1.79e+05	4.63e+05	1.79e+05	3.11e+05	2.00e+05	3.11e+05	3.42e+05	1.36e+05	3.43e+05
ABBV	4.80e+04	1.31e+05	4.77e+04	8.31e+04	6.01e+04	8.34e+04	9.29e+04	3.99e+04	9.38e+04
PM	3.60e+04	9.34e+04	3.59e+04	6.38e+04	3.80e+04	6.36e+04	7.06e+04	2.41e+04	7.06e+04
BAC	1.17e+04	4.38e+05	1.17e+04	4.06e+04	3.80e+05	4.07e+04	6.50e+04	3.31e+05	6.50e+04
CSCO	1.90e+04	3.98e+05	1.85e+04	5.88e+04	3.21e+05	5.57e+04	9.09e+04	2.57e+05	8.72e+04
KO	1.16e+04	1.97e+05	1.14e+04	3.45e+04	1.52e+05	3.35e+04	5.10e+04	1.19e+05	4.98e+04
ORCL	1.95e+04	2.69e+05	1.92e+04	5.25e+04	2.03e+05	5.26e+04	7.44e+04	1.58e+05	7.56e+04
PFE	1.49e+04	2.68e+05	1.50e+04	4.31e+04	2.12e+05	4.30e+04	6.40e+04	1.70e+05	6.39e+04
VZ	2.04e+04	2.42e+05	2.04e+04	5.62e+04	1.69e+05	5.70e+04	7.95e+04	1.22e+05	8.09e+04

**Table 5.** Stocks' average daily class distribution for the test set, computed across the 3-year analysis period, for HΔτ∈{10,50,100}.

	H10			H50			H100		
Ticker	Down	Stable	Up	Down	Stable	Up	Down	Stable	Up
CHTR	3.47e+04	4.49e+04	3.20e+04	4.87e+04	1.73e+04	4.57e+04	5.22e+04	9.96e+03	4.94e+04
GOOG	1.76e+05	3.02e+05	1.63e+05	2.72e+05	1.05e+05	2.63e+05	2.95e+05	5.42e+04	2.91e+05
GS	4.54e+04	5.19e+04	4.53e+04	6.45e+04	1.33e+04	6.48e+04	6.79e+04	6.62e+03	6.81e+04
IBM	5.82e+04	7.68e+04	5.90e+04	8.51e+04	2.42e+04	8.47e+04	9.01e+04	1.57e+04	8.82e+04
MCD	5.12e+04	5.91e+04	5.14e+04	7.20e+04	1.79e+04	7.18e+04	7.57e+04	1.08e+04	7.52e+04
NVDA	1.09e+05	7.34e+04	1.10e+05	1.37e+05	1.78e+04	1.37e+05	1.41e+05	1.12e+04	1.40e+05
AAPL	2.71e+05	3.50e+05	2.70e+05	3.75e+05	1.39e+05	3.76e+05	3.98e+05	9.35e+04	4.00e+05
ABBV	4.45e+04	8.43e+04	4.45e+04	7.09e+04	3.16e+04	7.08e+04	7.71e+04	1.99e+04	7.63e+04
PM	4.57e+04	8.92e+04	4.66e+04	7.62e+04	3.01e+04	7.53e+04	8.25e+04	1.85e+04	8.05e+04
BAC	1.88e+04	5.93e+05	1.88e+04	6.37e+04	5.03e+05	6.40e+04	1.02e+05	4.26e+05	1.02e+05
CSCO	4.82e+04	6.26e+05	4.84e+04	1.47e+05	4.29e+05	1.47e+05	2.10e+05	3.06e+05	2.07e+05
KO	2.54e+04	2.87e+05	2.55e+04	7.18e+04	1.94e+05	7.27e+04	9.93e+04	1.39e+05	1.00e+05
ORCL	4.22e+04	4.43e+05	4.18e+04	1.18e+05	2.92e+05	1.18e+05	1.65e+05	1.97e+05	1.65e+05
PFE	2.78e+04	3.51e+05	2.78e+04	7.64e+04	2.53e+05	7.64e+04	1.07e+05	1.92e+05	1.07e+05
VZ	4.59e+04	3.42e+05	4.63e+04	1.13e+05	2.09e+05	1.13e+05	1.43e+05	1.47e+05	1.43e+05

The first set (group 1, small-tick stocks) has cardinality equal to 6 and is made of CHTR, GOOG, GS, IBM, MCD and NVDA. At H10, the order of magnitude for the daily average number of samples for each label remains constant for the training, validation and test set, with only minor oscillations. At H50 and H100, the order of magnitude of representatives for classes ‘Up’ and ‘Down’ gradually increases, highlighting a more pronounced imbalance towards the two ‘active’ classes. This pattern can be detected in the training, validation and test set. The second group of stocks (group 2, medium-tick stocks) has cardinality equal to 3 and is made of AAPL, ABBV and PM. In this case, the order of magnitude of labels' representatives remains stable across horizons and for the training, validation and test set. Lastly, the third group of stocks (group 3, large-tick stocks) has a cardinality equal to 6 and is made of BAC, CSCO, KO, ORCL, PFE and VZ. The order of magnitude of representatives for the ‘Stable’ class is higher than the one for classes ‘Down’ and ‘Up’ at H10, while a stability is gradually matured moving to horizons HΔτ∈{50,100}. We highlight that this behavior is diametrically opposed to the one detected for group 1. As already underlined for the other two groups of assets, also in this case, the described pattern remains constant for the training, validation and test set.

## 5. Methods

From a practical perspective, this paper aims to provide a straightforward way to estimate a given stock's predictability based on its LOB microstructural properties. This aim can be achieved by splitting the research process into two steps: (i) extract and classify the microstructural properties of a heterogeneous set of stocks (see section 6); (ii) accomplish the forecasting task on each of them and review the obtained results in relation to the outcomes of the previous step.

### 5.1. LOBFrame architecture

**Figure 2.**
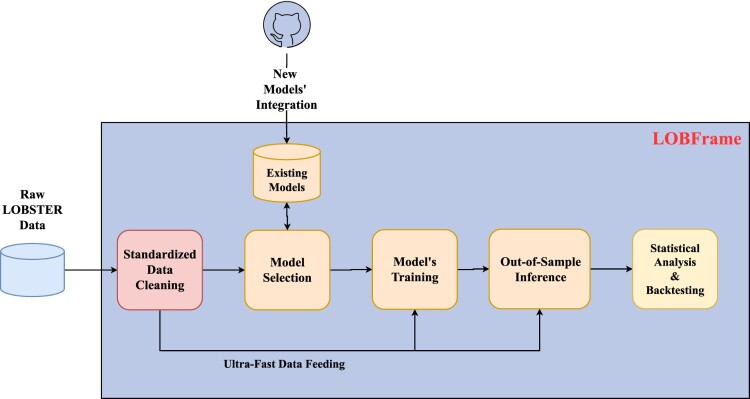
Pictorial representation of LOBFrame. This framework facilitates the Limit Order Book (LOB) forecasting practice through a seamless pipeline that includes data cleaning, standardized preprocessing, model selection, and integration of both existing and novel forecasting models, and ultra-fast data feeding. It enables comprehensive model training, out-of-sample inference, statistical analysis, and robust backtesting, offering the academic and practitioner communities a benchmark tool for advancing research and standardizing practices in the field. The use of distinct colors in the diagram highlights logically separated stages of the pipeline.

To perform the forecasting task, we release ‘LOBFrame’ (see figure 2), a novel, open-source code base, which presents a renewed way to process large-scale LOB data. This framework integrates all the latest cutting-edge insights from related scientific research into a cohesive system. Its strength lies in the comprehensive nature of the implemented pipeline, which includes the data transformation and processing stage, an ultra-fast implementation of the training, validation, and testing steps, as well as the evaluation of the quality of a model's outputs through trading simulations.^4^ Moreover, it offers flexibility by accommodating the integration of new models, ensuring adaptability to future advancements in the field. This contributes to the establishment of best practices in the field and fosters a more rigorous approach to forecasting model validation.

This paper integrates two crucial interconnected aspects: (i) the engineering effort behind the development of ‘LOBFrame’, and (ii) the valuable insights it enables within the field of market microstructure research. One challenge in fully conveying the strengths of LOBFrame lies in the necessity of selecting a specific forecasting model to demonstrate its capabilities. While this is essential for empirical analysis, it may inadvertently suggest that the framework is tied to a particular model, potentially overshadowing its broader applicability. To clarify, LOBFrame is model-agnostic—its strength lies not only in facilitating robust forecasting, but also in its ability to accommodate diverse predictive models and seamlessly adapt to future advancements. This adaptability ensures that it remains relevant as deep learning methodologies evolve. The framework is designed to be both flexible and accessible, making it a valuable tool for both academic research and industry applications. Researchers benefit from an open-source, modular infrastructure that facilitates experimentation and collaboration, while practitioners gain a practical solution for evaluating and deploying forecasting models in real-world trading environments.

The robustness of LOBFrame has been further validated in in the work by Briola *et al.* (2025), where the framework was applied across multiple forecasting models, consistently revealing fundamental microstructural patterns independent of model-specific characteristics. This underscores its reliability as a standardized tool for market microstructure research, providing consistent and actionable results across a variety of methodologies. By offering an open-source, flexible, and extensible pipeline, LOBFrame establishes itself as a benchmark resource for researchers and practitioners seeking to explore and advance LOB forecasting.

### 5.2. Deep learning model benchmark: DeepLOB

**Figure 3.**
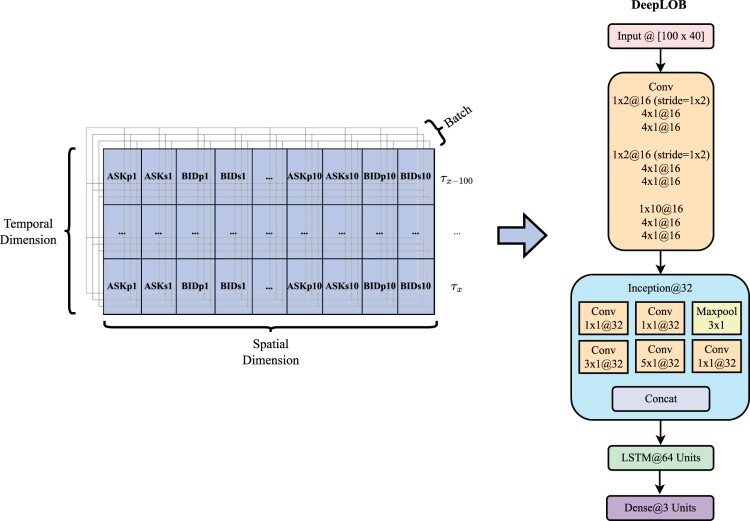
Pictorial representation of an input batch for the DeepLOB model (left-hand side of the Figure), and of the architecture itself (right-hand side of the Figure).

Results discussed in the current paper come from the usage of a state-of-the-art model in literature: DeepLOB (Zhang *et al.* 2019).^5^ This architecture mainly relies on two well-known deep learning modules: it exploits (i) the power of convolutional neural networks (CNNs) to model inter-levels, spatial LOB's dynamics (LeCun *et al.* 1998, O'Shea and Nash 2015, Albawi *et al.* 2017); and (ii) the memory of the LSTM module to handle the temporal dimension of the input (Hochreiter and Schmidhuber 1997, Van Houdt *et al.* 2020). For a detailed overview of the architecture, the reader is referred to the original work by Zhang *et al.* (2019), while, in this Section, our efforts are towards providing the intuition behind the model. The main idea of using CNNs is to automate the feature extraction process in a notoriously noisy and with low signal-to-noise ratio context (Briola *et al.* 2021) such as the one provided in LOB, without any strong initial assumption. Indeed, weights are learned during inference, and derived features (i.e. learned from the training set) are data-adaptive. The LSTM layer, on the other side, is used to capture residual time dependencies among the resulting features. It is worth underlining that short time dependencies are already captured by the convolutional layers, which take LOB snapshots as inputs (see figure 3). To train, validate and test the DeepLOB model, we design a high-performance data loader, which samples mini-batches of size 32 (as per in the original model's implementation), each made of inputs with size 100×40. Dimension 100 (i.e. the temporal dimension) represents the history length and corresponds to the number of historically consecutive LOB updates constituting each sample. Dimension 40, instead, is the number of spatial constituents for each LOB's snapshot (see equation (1)). The sampling process differs for the training, validation, and test sets. During training, the (sub)-sampling is random and balanced. From each trading day, we detect the number of samples for the less represented class and (i) if this value is ≥5000, then we sample 5000 random representatives (a representative is a 100×40 input) for each of the three classes (see equation (2)), otherwise, (ii) if this value is <5000, then we sample a number of random representatives for each class which is equal to the number of samples for the less represented class.

During validation and test stages, we still sample batches with a size of 32, but they are always sequential and cover the totality of data in the two sets. In line with the related literature (Zhang *et al.* 2019), the model is always trained for a maximum number of epochs equal to 100, with patience equal to 15 epochs. We use a modified version of the Adam optimizer (Kingma and Ba 2014) with decoupled weight decay (Loshchilov and Hutter 2017), commonly known as ‘AdamW’. Following the latest applied research findings (Brown *et al.* 2020, Karpathy 2024), we use a learning rate equal to $6\times 10^{-5}$, a ${\text{β}}_{1}$ decay rate equal to 0.9 and a ${\text{β}}_{2}$ decay rate equal to 0.95. The choices of values for these parameters are determined by the training pipeline described above, which is different from the one proposed in the original work (Zhang *et al.* 2019) and relies on a smaller number of training samples to reduce the model's exposition to the noise characterizing the LOB. The entire framework described in this paper is coded in Python using the PyTorch deep learning library (Paszke *et al.* 2019). A total number of 135 experiments have been run on the University College London Computer Science Department's High-Performance Computing Cluster (UCL 2024) for a cumulative GPU runtime of 959 hours, 16 minutes and 27 seconds. Six different types of GPUs have been used: (i) NVIDIA GeForce GTX 1080 Ti; (ii) NVIDIA GeForce RTX 2080 Ti; (iii) NVIDIA TITAN X (Pascal); (iv) NVIDIA TITAN Xp; (v) Tesla V100-PCIE-16GB; and (vi) Tesla V100-PCIE-32GB.

## 6. Microstructural priors

In this section, we investigate stocks' foundational microstructural properties, focusing on the interplay between tick size and bid-ask spread, liquidity at the best levels, and the structural organization of the Limit Order Book (LOB). Through this analysis:

- We identify a practical classification standard for tick-based stock categories, identifying unique behavioral patterns across small-, medium-, and large-tick stocks and revealing the macro-stability of spread distributions over the 3-year analysis period.
- We introduce the ‘actual LOB depth’ metric to quantify the sparsity of the LOB and uncover heterogeneous behaviors across stock categories, with large-tick stocks displaying more homogeneous structures and small-tick stocks showing higher variability.
- We analyze the relationship between physical time and tick-time, emphasizing the practical implications of varying trading activity levels across stock categories, with distinct clustering patterns for different prediction horizons.

As a first microstructural property, we study the relationship between the stocks' average spread 〈σ〉 and the tick size *θ* across the 3-year analysis period. In literature, a stock is differently classified based on a general definition which establishes that if 〈σ〉≫θ, than the asset is a small-tick stock, if 〈σ〉≃θ, than it is a large-tick stock (Bouchaud *et al.* 2018). Even if widespread, this definition is not quantitative and possibly too restrictive to characterize the more nuanced behavior of stocks traded in the NASDAQ exchange.

In this paper, we provide a practical classification which establishes that if (i) 〈σ〉⪆3θ, we are dealing with a small-tick stock; if (ii) 〈σ〉≲1.5θ, we are dealing with a large-tick stock; if (iii) 1.5θ≲〈σ〉≲3θ, we are dealing with a medium-tick stock. In this way, we impose quantitative boundaries for stock classification that allow the introduction of an extra family (i.e. medium-tick stocks) that groups ‘borderline’ assets. This category was previously identified by Bonart (2017) and Bouchaud *et al.* (2018). Considering that we analyse 3 years of data, one of the previously mentioned conditions should remain valid for at least 2 of the 3 considered years. Looking at table 6, we have 6 representatives of small-tick stocks (i.e. CHTR, GOOG, GS, IBM, MCD, NVDA), 3 representatives of medium-tick stocks (i.e. AAPL, ABBV, PM) and 6 representatives of large-tick stocks (i.e. BAC, CSCO, KO, ORCL, PFE, VZ). It is evident that, for small-tick stocks, the yearly average spread is subject to non-negligible fluctuations, while medium- and large-tick stocks are more stable across years. As we will point out several times in this paper, specific properties of small-, medium- and large-tick stocks highly impact their predictability.

**Table 6.** The 15 small-, medium- and large-tick stocks that we include in our analysis, along with their mean price and mean bid-ask spread during 2017, 2018 and 2019.

	2017		2018		2019		
	Mean	Mean	Mean	Mean	Mean	Mean	
Ticker	price [$]	spread [$]	price [$]	spread [$]	price [$]	spread [$]	Size
CHTR	343.65	0.2869	312.61	0.3475	394.76	0.2206	small
GOOG	934.44	0.4362	1099.33	0.7898	1186.57	0.5511	small
GS	232.82	0.0965	223.35	0.1111	204.19	0.0759	small
IBM	157.90	0.0362	140.23	0.0444	137.94	0.0316	small
MCD	146.71	0.0321	166.39	0.0542	198.29	0.0531	small
NVDA	144.12	0.0437	233.82	0.0844	172.13	0.0500	small
AAPL	151.97	0.0145	190.11	0.0223	208.62	0.0190	medium
ABBV	71.59	0.0211	94.98	0.0422	76.86	0.0212	medium
PM	110.78	0.0231	86.96	0.0293	81.89	0.0240	medium
BAC	24.69	0.0109	29.31	0.0109	29.40	0.0105	large
CSCO	33.20	0.0106	44.25	0.0110	51.31	0.0107	large
KO	44.11	0.0112	45.84	0.0116	51.00	0.0111	large
ORCL	46.51	0.0115	47.89	0.0117	54.05	0.0111	large
PFE	33.93	0.0111	39.85	0.0114	39.99	0.0109	large
VZ	48.30	0.0119	52.80	0.0121	57.92	0.0112	large

**Figure 4.**
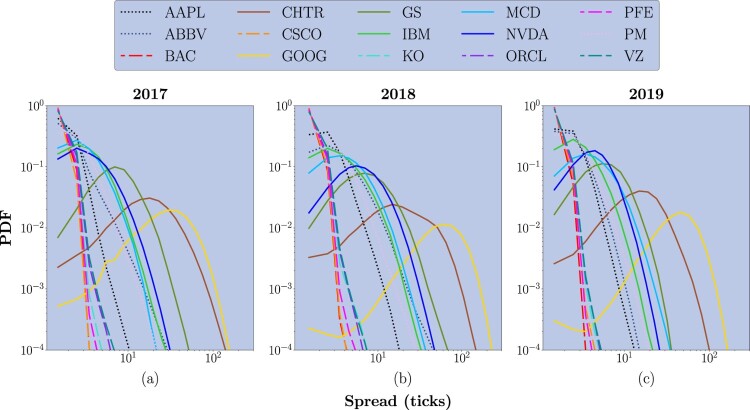
PDF of the spread (expressed in number of ticks) for the 15 stocks of interest, in the 3-year analysis period.

Extending the previous analysis, in figure 4, we report the PDF of the spread (expressed in number of ticks) for each considered stock. As one can notice, distributions are different for small-, medium- and large-tick stocks, defining evident behavioral clusters. For large-tick stocks, distributions are peaked at an average value of 1.5 ticks (extremely close to the minimum spread allowed of 1 tick), with rare openings to larger realizations. This finding is consistent across the 3 years. It is worth noting that, from a practical perspective, tighter spreads are beneficial for traders looking for stocks allowing to enter and exit positions quickly and with minimal impact on the transaction costs. For medium-tick stocks, distributions are peaked slightly over the minimum spread: during 2017, the average peak value is equal to 1.50 ticks; during 2018, the average peak value is equal to 2.50 ticks; while, during 2019, the average peak value is equal to 1.83 ticks. Notably, these distributions express more significant variations than those describing large-tick stocks. Among these assets, AAPL is characterized by a distribution with a shape more similar to that of large-tick stocks, while ABBV and PM show a behavior more similar to that of small-tick stocks. This result is expected since, by definition, medium-tick stocks are ‘borderline’ assets characterized by behavioral patterns that do not clearly belong to the class of small- nor large-tick stocks. Lastly, small-tick stocks show consistently broader distributions. In this family, we distinguish two different subsets of assets: the first one is made of CHTR, GOOG, and GS, while the second one is made of IBM, MCD, and NVDA. Distributions characterizing the first subset have an average peak of 18.16 ticks in 2017, 27.50 ticks in 2018 and 22.60 ticks in 2019. Distributions characterizing the second subset, instead, have an average peak equal to 2.50 ticks in 2017, 3.83 ticks in 2018 and 3.50 ticks in 2019. In both cases, small-tick stocks express more significant variances than large-tick stocks, suggesting less frequent trading activity or larger orders that could move the market (Bouchaud *et al.* 2018) and, consequently, an higher exposition to market impact for actors placing trades. It is worth noting that, for each class of stocks, the shape of the spread's distribution remains consistent over the three years, suggesting an overall macro-stability.

**Figure 5.**
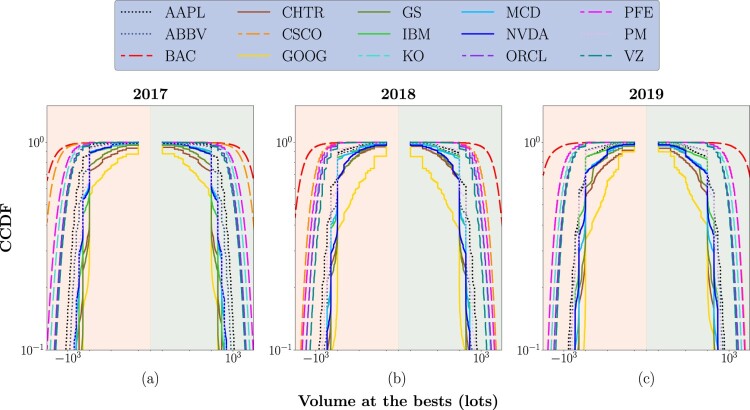
CCDF of the volumes available at the best quotes for the 15 stocks of interest, in the 3-year analysis period.

A second microstructural aspect to investigate concerns the liquidity at the best levels. Indeed, once the impact of stocks' tick size on the potential costs related to fast trading is clarified, it is relevant to study the CCDF of volumes available at the best quotes to understand if there is the necessary liquidity to perform such an activity. Figure 5 reports the results of this analysis. The *x*-axis utilizes a symmetric log-scale to study both the ask side (negative part, red area) and the bid side (positive part, green area) of the LOB, while underlying the broadness of distributions. As one can notice, distributions are roughly symmetric for the two sides; also, in this case, a behavioral clustering directly dependent on the tick size of the stocks is evident. Distributions characterizing large-tick stocks are significantly wider, highlighting a condition of higher liquidity at best quotes. Even if not visible in figure 5, as explained by Bouchaud *et al.* (2018), it is relevant to underline that in the case of large-tick stocks, the volume of the queues decreases before transactions since liquidity takers rush to take the remaining volumes before it disappears. This phenomenon provides more information on the direction of future price changes, potentially contributing to an improved forecast accuracy of deep learning models. One more time, it is possible to highlight the ‘borderline’ behavior of medium-tick stocks. They exhibit distributions that fall in the middle between the ones characterizing large- and small-tick stocks. Lastly, the curves characterizing small-tick stocks are the steepest, highlighting an overall condition of lower liquidity and potentially higher volatility. Also in this case, even if not immediately visible from figure 5, as explained by Bouchaud *et al.* (2018), it is relevant to underline that the volume at the best quote increases immediately before being hit by a market order, indicating that liquidity takers choose to submit their orders when the opposite volume is relatively high.

As pointed out in the work by Wu *et al.* (2021), the LOB representation adopted in the current paper (see equation (1)), which is commonly referred to as ‘compressed representation’ (Wu *et al.* 2021), presents a major drawback: its spatial structure is not homogeneous (see figure 1) since there is no assumption for adjacent price levels to have fixed intervals, while only a monotonic order is guaranteed (Wu *et al.* 2021). This representation is prone to dramatic changes due to occasional price-level shifts, significantly impacting predictability when treated as input for deep learning models. Indeed, in the work by Wu *et al.* (2021), the authors underline that one of the main assumptions in deep learning is that signals from the same channel (or input dimension) are from the same source. In our case, a ‘level’ is an artifact strictly related to a single snapshot of the LOB and it is not associated with a constant source, especially when its information shifts due to aggressive orders. To measure stocks' exposure to this issue, we compute a metric defined as the ‘actual LOB depth’ (Ξ). Given a snapshot L(τ), this measure is computed for the two sides of the market as follows:

$$\begin{array}{rl} \Xi_{\tau }^{\mathrm{Ask}} & \;=\frac{p_{10}^{\mathrm{ask}}(\tau )-p_{1}^{\mathrm{ask}}(\tau )}{\theta }\;\mathrm{ask}\;\mathrm{side},\\ \Xi_{\tau }^{\mathrm{Bid}} & \;=\frac{p_{1}^{\mathrm{bid}}(\tau )-p_{10}^{\mathrm{bid}}(\tau )}{\theta }\;\mathrm{bid}\;\mathrm{side}\;. \end{array}$$

**Figure 6.**
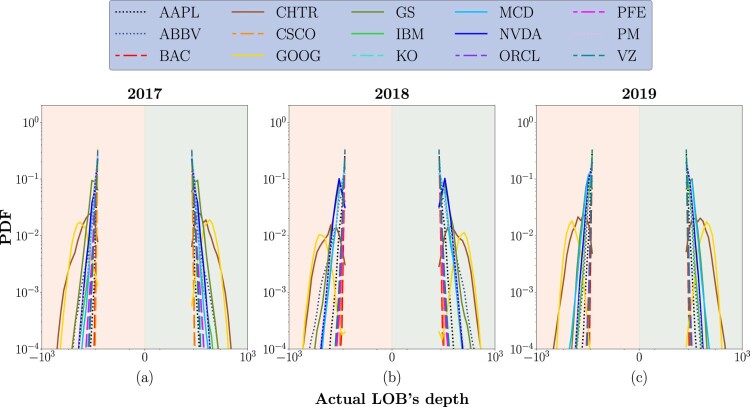
PDF of the ‘actual LOB depth’ (Ξ) for the 15 stocks of interest, in the 3-year analysis period.

In figure 6, we report the PDF for $\Xi^{\mathrm{Bid}}$ and $\Xi^{\mathrm{Ask}}$ for each stock, across the 3-year period of analysis.

Even if less evident than in previous analyses, detecting separate clusters of stocks displaying a different behavior is still possible. One more time, distributions are roughly symmetric for the two sides of the LOB. The likelihood of having a homogeneous spatial structure across different levels is higher for large-tick stocks. In this case, distributions have an average peak equal to 9.50 price levels (slightly more than the minimum allowed distance between the two extreme levels of the LOB) for both the ask and bid side across all the 3-year analysis period. The same behavior is detected for medium-tick stocks even if distributions are slightly wider, especially for PM and ABBV, suggesting a higher likelihood of extreme events. Lastly, when analyzing small-tick stocks, it is useful to divide them into two separate subsets as we did above: the first set is made of CHTR, GOOG and GS, while the second is made of IBM, MCD and NVDA. Distributions characterizing the first subset have an average peak equal to 23.10 and 23.00 price levels for the bid and ask side, respectively, in 2017, 37.50 and 40.83 in 2018, and 31.00 and 26.66 in 2019. Distributions characterizing the second subset have an average peak equal to 9.50 for both bid and ask sides in 2017, 11.33 for both sides in 2018, and 11.33 and 10.50 in 2019. In this case, distributions are much wider than those characterizing large-tick stocks, underlying a higher likelihood of heterogeneous spatial structure across different levels of the LOB. This means that the corresponding stocks are characterized by a sparse LOB structure with empty levels, potentially inflating the inner representation of deep learning models.

In addition to these foundational microstructural properties, many derived ones have been recently introduced. Despite the goal of all of them is digging into a specific (sub)-aspect of the LOB microstructural structure, it is easily demonstrable that most of them can be directly mapped to one or more of the fundamental quantities introduced earlier in this Section. An example is the ‘information richness’ (IR) score (Kolm *et al.* 2023). In the original paper, the authors claim it is a measure of stocks' predictability; this is only partially true. As we empirically show in Appendix 1, there is a direct mapping between the IR score of a stock and its tick size; consequently, the tick size itself could be used as a proxy measure of a stock's predictability.

So far, in all the analyses, we have always defined the time in terms of number of LOB updates (i.e. ‘tick time’). This means that, for different stocks, there is a different mapping between physical time and tick-time. This aspect constitutes an issue from the point of view of practitioners who are not interested in the forecasts as the result of a mere academic exercise, but are mainly focused on their actual practicability in real-world scenarios. In table 7, we report the average probability (computed across the 3-year analysis period) that the number of updates characterizing the three horizons HΔτ∈{10,50,100}, happens in a physical time (i) <1 second (s), (ii) ≥1 and <10 seconds, or (iii) ≥10 seconds. For each HΔτ∈{10,50,100}, the probabilities of the three cases sums to 1. As one can notice, for all the stocks, except MCD (small-tick stock) and ABBV (medium-tick stock), 10 LOB's updates are more likely to happen in a physical time <1 s. 50 LOB's updates, instead, are more likely to happen in a physical time ≥1 s ∧ <10 s except that for CHTR (small-tick stock), MCD (small-tick stock) and AAPL (medium-tick stock). The case of AAPL is particularly notable since it is characterized by a remarkably more frequent trading activity than observed in all the other assets. Lastly, H100 represents the scenario where behavioral clustering is more evident among different classes of stocks. For small-tick stocks, 100 LOB's updates are more likely to happen in a physical time ≥10 s. The only two exceptions are GOOG and NVDA, which are characterized by higher trading activity. For medium-tick stocks, ABBV and PM, as per all the other microstructural analyses, show a behavior which is comparable to the one of small-tick stocks, while AAPL has a behavior more similar to the one of large-tick stocks. Indeed, for this last class of stocks, 100 LOB's updates are always more likely to happen in a physical time ≥1 s ∧<10 s, delineating a trading activity which is higher than the one of small- and medium-tick stocks.

**Table 7.** Average probability (computed across the 3-year analysis period) that the number of updates characterizing the three horizons HΔτ∈{10,50,100}, happens in a physical time (i) <1 second; (ii) ≥1 second and <10 seconds; or (iii) ≥10 seconds.

	<1 s			>=1 s & <10 s			>=10 s		
Ticker	H10	H50	H100	H10	H50	H100	H10	H50	H100
CHTR	0.46	0.06	0.01	0.41	0.36	0.18	0.13	0.58	0.81
GOOG	0.76	0.32	0.14	0.18	0.53	0.56	0.06	0.15	0.30
GS	0.51	0.05	0.01	0.39	0.56	0.29	0.10	0.39	0.70
IBM	0.57	0.07	0.01	0.35	0.65	0.40	0.08	0.28	0.59
MCD	0.00	0.00	0.00	0.00	0.00	0.00	1.00	1.00	1.00
NVDA	0.72	0.24	0.06	0.18	0.68	0.73	0.10	0.08	0.21
AAPL	0.92	0.55	0.23	0.04	0.41	0.73	0.04	0.04	0.04
ABBV	0.00	0.00	0.00	0.31	0.63	0.40	0.69	0.37	0.60
PM	0.63	0.07	0.01	0.32	0.69	0.42	0.05	0.24	0.57
BAC	0.78	0.43	0.30	0.12	0.47	0.55	0.10	0.10	0.15
CSCO	0.80	0.45	0.24	0.13	0.46	0.58	0.07	0.09	0.18
KO	0.68	0.33	0.14	0.23	0.49	0.51	0.09	0.18	0.35
ORCL	0.75	0.39	0.18	0.17	0.45	0.56	0.08	0.16	0.26
PFE	0.72	0.38	0.17	0.19	0.48	0.54	0.09	0.14	0.29
VZ	0.73	0.38	0.16	0.19	0.48	0.57	0.08	0.14	0.27

## 7. Results

In this Section, we report the results of our analysis, in particular concerning (i) the assessment of the DeepLOB model performance for mid-price changes direction forecast using traditional machine learning metrics; and (ii) the introduction of a novel, cutting-edge strategy-oriented methodology that computes the probability of correctly predicting a transaction using the model's forecasts. In all the experiments, we assess the behavior of the three classes of stocks (i.e. small-, medium- and large-tick stocks) at 3 predictions horizons HΔτ∈{10,50,100}, at different confidence levels (i.e. adopting various probability thresholds). The classification of stocks into small-, medium-, and large-tick reflects distinct microstructural behaviors, as detailed in section 6. The goal is to evaluate the forecasting performances in different scenarios and link them to the microstructural properties of the stocks and the complex underlying LOB dynamics. For instance, stocks with small tick sizes tend to exhibit broader spread distributions (figure 4), and lower liquidity at best levels (figure 5): these microstructural characteristics influence forecasting performance by affecting the signal-to-noise ratio and the reliability of observed patterns in LOB data.

### 7.1. Assessing model's forecast performances using traditional machine learning metrics

This section provides a detailed evaluation of the forecasting performance of the DeepLOB model across different classes of stocks. The focus is on analyzing the confusion matrices and derived performance metrics to characterize the model's predictive accuracy and error patterns. The main findings are summarized as follows:

- Models trained on small- and medium-tick stocks show significant reciprocal misclassification between extreme classes (i.e. −1 and 1), with higher misclassification rates at longer prediction horizons.
- Large-tick stocks consistently exhibit superior predictive performance, with fewer misclassifications and robust results across all horizons, as reflected in both confusion matrices and derived metrics.
- The application of probability thresholds enhances predictive accuracy for all stock classes, with large-tick stocks demonstrating both higher accuracy and greater resilience in maintaining a substantial proportion of usable forecasts even at stricter thresholds.

**Figure 7.**
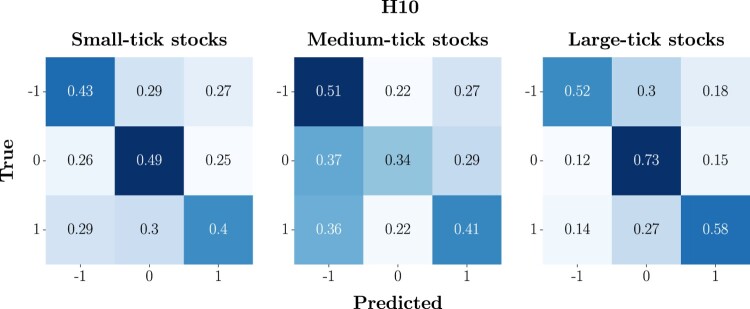
Average confusion matrices at H10. To obtain these compact representations, we firstly compute individual confusion matrices for each stock over the 3-year analysis period, aggregating them into a list based on the class (i.e. small-, medium- and large-tick stocks). The average matrix is obtained by summing these matrices and dividing by their count, thus reflecting overall performance metrics. This average is finally normalized row-wise, turning counts into proportionate metrics of predictive accuracy and class-specific performance. The final normalized matrix succinctly visualizes the model's average effectiveness in classifying mid-price changes directions, during the period of interest.

To assess the forecasting performances of the DeepLOB model, we analyse the confusion matrices computed for each class of stocks – small-, medium- and large-tick stocks – at 3 predictions horizons HΔτ∈{10,50,100}, across the 3-year analysis period. In figure 7, we show the average confusion matrix for each class of stocks at H10. We observe that models trained on small- and medium-tick stocks demonstrate a non-negligible frequency of reciprocal misclassifications between the extreme classes (−1 and 1), corresponding to a ‘Down’ and ‘Up’ movement, respectively. Specifically, for small-tick stocks, the 29% of true class 1 is misclassified as class −1, and the 27% of true class −1 is misclassified as class 1. Medium-tick stocks exhibit a similar pattern with a slight increase in misclassification for true class 1 as class −1 (i.e. 36%). Conversely, for large-tick stocks, the model's predictive performance is markedly distinct with a stronger ability to correctly classify the two extreme classes, and most of the errors concentrated towards their misclassification as 0.

**Figure 8.**
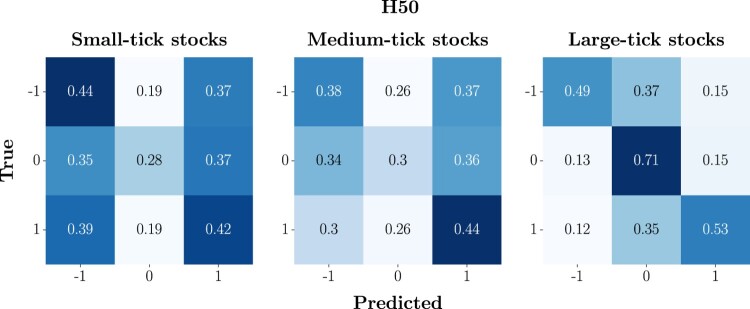
Average confusion matrices at H50. To obtain these compact representations, we firstly compute individual confusion matrices for each stock over the 3-year analysis period, aggregating them into a list based on the class (i.e. small-, medium- and large-tick stocks). The average matrix is obtained by summing these matrices and dividing by their count, thus reflecting overall performance metrics. This average is finally normalized row-wise, turning counts into proportionate metrics of predictive accuracy and class-specific performance. The final normalized matrix succinctly visualizes the model's average effectiveness in classifying mid-price changes directions, during the period of interest.

In figure 8, we report the average confusion matrix for each class of stocks, across the 3-year analysis period, at H50. In this case, we observe that, compared to what happens at H10, models trained on small- and medium-tick stocks have a higher tendency to mix the extreme classes (−1 and 1), which, we stress again, anticipate a ‘Down’ and ‘Up’ movement. For small-tick stocks, we observe that the 39% of the true class 1 instances are misclassified as class −1, and the 37% of true class −1 instances are mistaken for class 1. Medium-tick stocks show a comparable trend, with slightly more misclassification of extreme classes to the central one. On the other hand, for large-tick stocks, the model performance remains similar to the one observed at H10.

**Figure 9.**
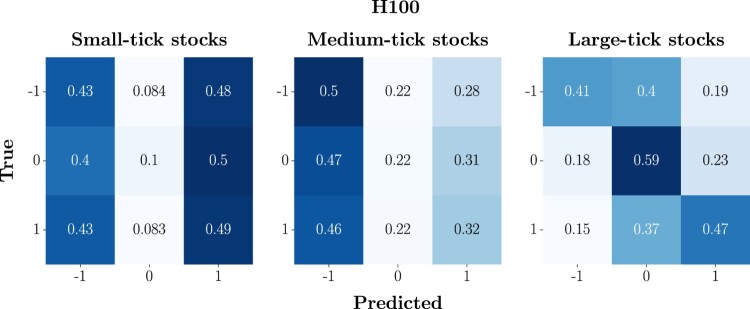
Average confusion matrices at H100. To obtain these compact representations, we firstly compute individual confusion matrices for each stock over the 3-year analysis period, aggregating them into a list based on the class (i.e. small-, medium- and large-tick stocks). The average matrix is obtained by summing these matrices and dividing by their count, thus reflecting overall performance metrics. This average is finally normalized row-wise, turning counts into proportionate metrics of predictive accuracy and class-specific performance. The final normalized matrix succinctly visualizes the model's average effectiveness in classifying mid-price changes directions, during the period of interest.

In figure 9, we report the average confusion matrix for each class of stocks, across the 3-year analysis period, at H100. In this case, we observe that models trained on small-tick stocks have an equal tendency to correctly classify and misclassify the two extreme classes. In addition to this, in line with what is observed for H50, there is a remarkable tendency to classify class 0 as −1 or 1, further incrementing the probability of critical errors. A similar scenario is detected for medium-tick stocks, while, for large-tick stocks, one more time, the model's performance remains consistent with the one observed at H10 and H50.

**Figure 10.**
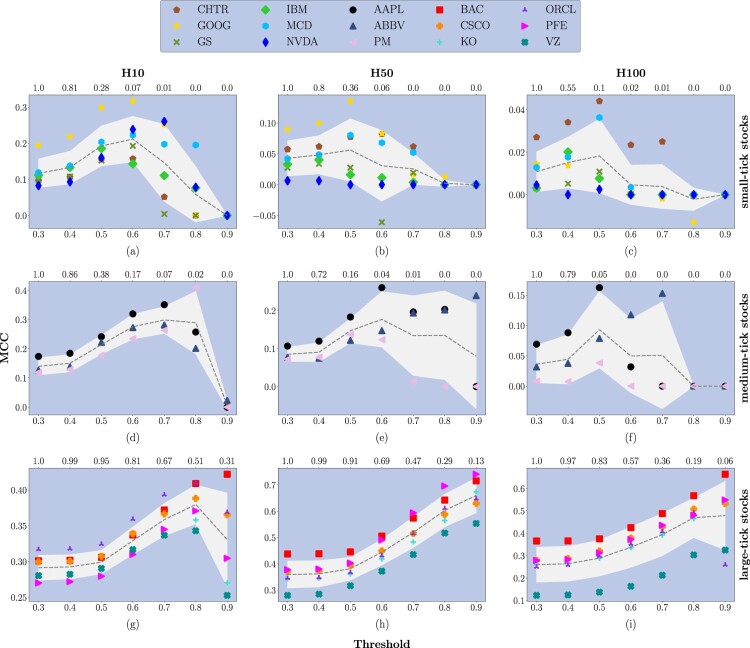
Average Matthews Correlation Coefficient (MCC). Results are organized according to the prediction horizons taken into account (see columns) and stocks' tick-size (see rows). Each plot contains three main pieces of information: (i) the model's performance changes applying different thresholds on the probabilities associated with each forecast (shown on the bottom of the *x*-axis); (ii) the average percentage amount of remaining data after using the threshold (shown on the top of the *x*-axis); (iii) the performance average pattern and the corresponding standard deviation (shown through the gray line and shadows). All the average values and the standard deviations are computed by considering stocks with the same tick-size, spanning the 3-year analysis period.

Confusion matrices serve as the foundational instrument for presenting the behavior of predictive models in their broadest context. They provide a detailed breakdown of the model's forecasts, which helps in evaluating the performance across different scenarios. However, to gain a deeper insight into a model's capabilities and to make more nuanced assessments of its effectiveness, derived metrics are essential. These metrics offer a more granular view of the model's predictive accuracy and error tendencies, facilitating a comprehensive understanding of its strengths and limitations. By analyzing derived metrics, researchers and practitioners can better comprehend the potential of each model, enabling them to make informed decisions regarding its application and improvement. To assess the forecasting performances of the DeepLOB model we use the Matthews Correlation Coefficient (MCC) (Gorodkin 2004). It is a generalization of Pearson's correlation coefficient between actual and predicted classes; it takes values between −1 (in case of inverse prediction) and +1 (in case of perfect prediction), while a value of 0 indicates a random prediction. MCC is generally regarded as a balanced measure which can be used even if the classes are of very different sizes (Chicco 2017, Powers 2020). Figure 10 shows the average MCC computed on the 3-year analysis period for the different classes of stocks. Results are organized according to prediction horizons (see columns) and stocks' tick-sizes (see rows). Each plot contains three main pieces of information: (i) the model performance's changes by applying different probability thresholds (shown on the bottom of the *x*-axis); (ii) the average percentage amount of remaining data after using probability thresholds (shown on the top of the *x*-axis); (iii) the performance average trend (computed across stocks belonging to the same class) and the corresponding standard deviation (shown through the gray line and shadow, respectively). Looking at these results, it is worth highlighting the different scales on the *y*-axes for each horizon and each class of stocks.

Overall, we observe that the DeepLOB model exhibits greater misclassification rates for small-tick stocks at H50 and H100. This aligns with the findings in Section 6, where small-tick stocks have a significantly wider distribution of spread realizations (figure 4) and lower liquidity at best quotes (figure 5). These factors contribute to noisier market environments, reducing the model's ability to distinguish between price movement classes.

At H10, without the application of any threshold, the average MCC for *small-tick stocks* is 0.11, while the standard deviation is 0.04, with only one stock (GOOG) acting as an outlier with an average MCC value (computed across the 3-year period of analysis) equal to 0.19. We observe that, for thresholds ≤0.6, there is an increasing pattern in the average performance. This behavior is associated with a rapid decrease in the average percentage of data used for the metric's computation. The average performance decreases for thresholds >0.6, and only an average percentage of data <1% is used for the metric's computation. Similar findings are detected for *medium-tick stocks*. At H10, without applying any threshold, the average MCC is 0.13, while the standard deviation equals 0.03. By increasing the threshold value, we observe an increase in performance and a decrease in the percentage of data used for the metric's computation. Such a decrease is smoother if compared to the one observed in small-tick stocks, but still relevant. Among medium-tick stocks, AAPL performs slightly better than other stocks, remarking an intra-class separation that we already observed from the point of view of microstructural properties in section 6. The scenario radically changes for *large-tick stocks*. At H10, without applying any threshold, the average MCC is 0.29 (18 units higher than the one characterizing small-tick stocks and 16 units higher than the one characterizing medium-tick stocks). At the same time, the standard deviation has a value equal to 0.017. In this case, a threshold-dependent increasing pattern is also evident, especially for values >0.5. However, unlike the other two classes of stocks, the average percentage of data used to compute the metric remains remarkably high. In this sense, the case of threshold equal to 0.9 is meaningful since the metric is still computed using the 31% of available forecasts, hence highlighting an enhanced strength of the signal associated with each forecast.

Moving to H50, we note that, without applying any threshold, the average MCC for *small-tick stocks* is 0.04, while the standard deviation is 0.029. Also in this case, GOOG acts as an outlier with an average MCC value (computed across the 3-year period of analysis) equal to 0.089. The same happens for NVDA, but in negative terms: in this case, the average MCC value equals 0.006. We remark that by varying the threshold, the average performance for small-tick stocks remains almost constant. In contrast, the decrease in the average percentage of data used for the metric's computation is comparable to that observed at H10. For *medium-tick stocks*, at H50, without applying any threshold, the average MCC is 0.085, while the standard deviation has a value of 0.019. An average growing pattern is detected for threshold values ≤0.6. In contrast, the average percentage of values used for metric computation decreases with the same velocity as in small-tick stocks. A larger standard deviation is detected for threshold values >0.6. AAPL always performs better than other stocks belonging to the same class. For *large-tick stocks*, at H50, without the application of any threshold, the average MCC is 0.36 (32 units higher than the one of small-tick stocks and 28 units higher than the one of medium-tick stocks). At the same time, the standard deviation has a value of 0.056. A clear average growing pattern is detected for all threshold values. In contrast, the average percentage of data used to compute the metric decreases considerably more than what happened at H10, remaining higher than the minimum reached by small- and medium-tick stocks. We remark that the difference between the average performance at threshold 0.9 and the one without threshold (i.e. threshold equals 0.3) equals 0.30.

Lastly, considering H100, we notice that, without applying any threshold, the average MCC for *small-tick stocks* is 0.01, while the standard deviation is 0.009. These results suggest that the model is producing random forecasts. The average performance remains almost constant, varying the threshold, while the decrease in the average percentage of data used for the metric's computation is the steepest if compared to the values at HΔτ∈10,50. For *medium-tick stocks*, at H100, without applying any threshold, the average MCC is 0.036, while the standard deviation has a value of 0.03. An average growing pattern is detected for threshold values ≤0.5, while the decrease in average percentage is as steeper as in small-tick stocks. A larger standard deviation is detected when the threshold value is ≤0.8, with AAPL stock performing, one more time, better than other class components. For *large-tick stocks*, at H100, without the application of any threshold, the average MCC is 0.26 (25 units higher than the one of small-tick stocks and 23 units higher than the one of medium-tick stocks). At the same time, the standard deviation has a value of 0.078. Differently from what happens for the other two classes of stocks, a clear average growing pattern is detected for threshold values ≤0.8, while the average percentage of data used to compute the metric decreases more than what happened at H50, remaining, however, higher than the minima reached for small- and medium-tick stocks. Overall, large-tick stocks demonstrate greater forecast stability across horizons, as evidenced by the lower variance in MCC. This robustness is strongly linked to their higher liquidity and less broad spread distributions, which provide a more stable market dynamics for the DeepLOB model to learn from.

**Figure A1.**
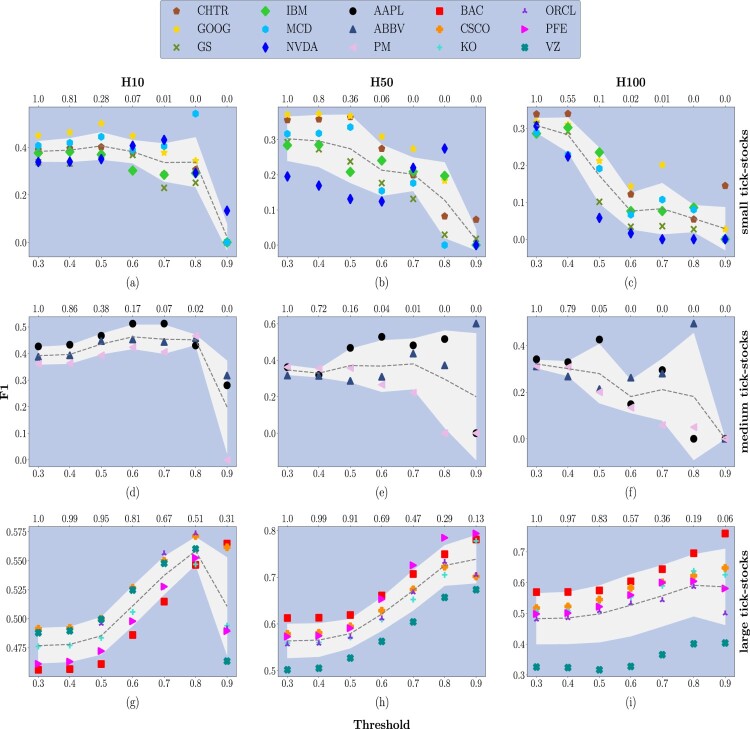
DeepLOB model's average F1 score. Results are organized according to the prediction horizons taken into account (see columns) and stocks' tick-size (see rows). Each plot contains three main pieces of information: (i) the model's performance changes applying different thresholds on the probabilities associated with each forecast (shown on the bottom of the *x*-axis); (ii) the average percentage amount of remaining data after using the threshold (shown on the top of the *x*-axis); (iii) the performance average pattern and the corresponding standard deviation (shown through the gray line and shadows). All the average values and the standard deviations are computed by considering stocks with the same tick-size, spanning the 3-year analysis period.

**Figure A2.**
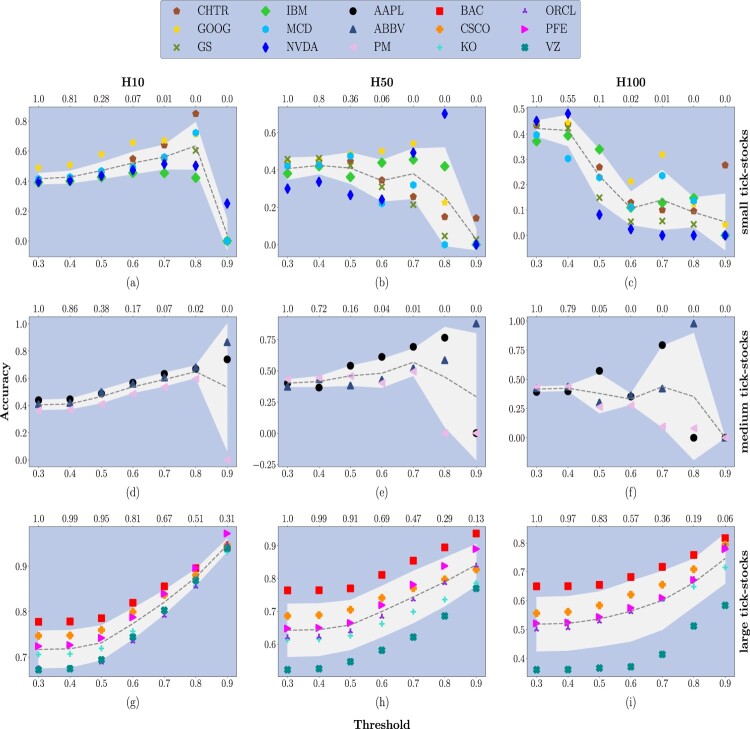
DeepLOB model's average accuracy score. Results are organized according to the prediction horizons taken into account (see columns) and stocks' tick-size (see rows). Each plot contains three main pieces of information: (i) the model's performance changes applying different thresholds on the probabilities associated with each forecast (shown on the bottom of the *x*-axis); (ii) the average percentage amount of remaining data after using the threshold (shown on the top of the *x*-axis); (iii) the performance average pattern and the corresponding standard deviation (shown through the gray line and shadows). All the average values and the standard deviations are computed by considering stocks with the same tick-size, spanning the 3-year analysis period.

The analysis reported in this Section is further deepened in Appendices 2 and 3, where we report (i) the year-wise MCC of the DeepLOB model at HΔτ∈{10,50,100}, for different confidence levels; (ii) the corresponding statistical significance; and (iii) a replica of the analysis in figure 10 for the F1 and accuracy score. The coherence of the results bolsters the robustness of the findings discussed earlier in this Section, highlighting that large-tick stocks exhibit a significant predictability rate across all the considered horizons. This is evidenced in figures A1 and A2, where we observe that these stocks achieve F1 score realizations greater than 0.45 without applying any thresholds and surpass 0.7 when probability thresholds are implemented. Similarly, accuracy scores exceed 0.7 without thresholds and reach over 0.9 with the application of probability thresholds. However, as detailed in section 7.2, achieving high scores on these traditional machine learning metrics does not necessarily translate into the generation of actionable trading signals. This distinction remarks the complexity of converting predictive accuracy into practical trading strategies.

### 7.2. On the practicability of model's forecasts

The analysis presented above offers significant insights into the DeepLOB model's performance at different horizons for different classes of stocks. However, further discussion is needed to understand the results from the perspective of the microstructural properties of the LOB. To do so, in this Section, we introduce a novel methodology to evaluate the practicability of forecasts. The main findings are summarized as follows:

- We demonstrate that for a signal to be considered tradable, the chronological positioning of correct forecasts is more critical than their sheer abundance.
- We show that, consistently with previously discussed results, using the newly introduced metric, large-tick stocks exhibit higher practicability compared to small- and medium-tick stocks.
- We find that the effectiveness of the newly introduced metric can be further enhanced by relaxing certain construction constraints.

**Figure 11.**
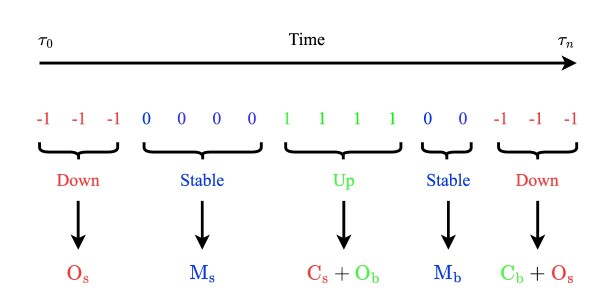
Pictorial representation of a chronologically sorted vector of forecasts. Following the mapping in equation (2), we derive a simplified strategy where $O_{p\in \{s/b\}}$ means ‘opening a new selling/buying position’, $M_{p\in \{s/b\}}$ means ‘maintaining an existing selling/buying position’, $C_{p\in \{s/b\}}$ means ‘closing an existing selling/buying position’.

**Figure 12.**
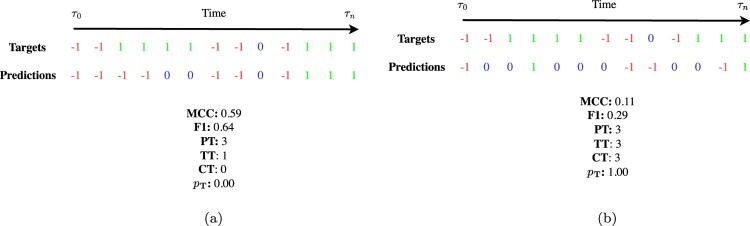
Transaction-related (PT,TT, CT, $p_{T}$) and machine learning metrics (MCC, F1) computed on two chronologically sorted vectors of forecasts and corresponding targets.

Despite the recent attempts made in state-of-the-art research papers (Zhang *et al.* 2019, Wood *et al.* 2021, Yin and Wong 2023), backtesting a trading strategy based on the outputs of a deep learning model by using historical data-only is not possible. Indeed, several assumptions are needed, including but not limited to (i) having the technical and infrastructural potential to record and process live data, produce forecasts and execute them in due time; (ii) being always executed; (iii) having a zero market-impact; (iv) having zero transaction costs. The combination of all or part of these assumptions ruins any attempt to produce a reliable backtest, and, indeed, it is different from what academics should try to achieve to bridge the gap with the practitioners' community. In this Section, we propose a strategy-oriented analysis of the model's forecasts, which is entirely assumption-free and fully immune to class imbalances. As an introductory example, let us consider a scenario where the mid-price changes' direction forecasts, which, in our case, are always chronologically sorted, are defined as per in figure 11. The direct mapping between predictions and trading actions would include (i) opening a selling position in correspondence of the first predicted mid-price ‘Down’ movement (i.e. $O_{s}$); (ii) maintaining the selling position in correspondence of the predicted mid-price ‘Stable’ period (i.e. $M_{s}$); (iii) closing the existing selling position (i.e. $C_{s}$), while opening a new buying position (i.e. $O_{b}$) in correspondence of the predicted mid-price ‘Up’ movement; (iv) maintaining the position in correspondence of the predicted mid-price ‘Stable’ period (i.e. $M_{b}$); and, (v) closing the existing buying position (i.e. $C_{b}$), while opening a selling position (i.e. $O_{s}$) in correspondence of the newly predicted mid-price ‘Down’ movement. By performing this simplified strategy, we would have opened 3 positions and closed 2 of them, overall completing 2 transactions (i.e. a transaction is completed when a position is successfully opened and later closed). Using forecasts, however, necessarily implies relying on their ‘correctness’. To contextualize this concept, let us consider the two examples of chronologically sorted vectors of forecasts presented in figure 12. For each of them, we report the MCC, the F1 score, and the following transactions-related metrics:

- The number of potential transactions (PT). Looking at figure 11, we remark that a transaction happens when one is able to open a position and then close it (i.e. $O_{s}\to C_{s}\;\vee \;O_{b}\to C_{b}$). In this context, we use the term ‘potential’ because transactions are counted on the targets' set.
- The total number of executed transactions (TT). This metric is computed in the same manner as PT, but on the predictions' set.
- The total number of correctly executed transactions (CT). This metric counts how many times a transaction executed on the predictions' set has a correspondence in the targets' set. In figure 12(a), we show an example where CT=0, due to discrepancies in the positions' entering/exiting points in the two sets.
- The probability $p_{T}$ to execute a correct transaction.

From the definitions provided above, it is evident that the set CT is given by the intersection of PT and TT sets; the probability to execute a correct transaction, $p_{T}$, is hence computed as follows:

(3) $$p_{T}=\frac{\mathrm{CT}}{\mathrm{PT}+\mathrm{TT}-\mathrm{CT}}.$$

We remark that, being our approach assumption-free, when we refer to ‘opening/closing a position’ and ‘executing a transaction’, we mean the model's capability to accurately identify an optimal entry point for initiating or concluding a trade, either as separate actions or together, respectively.

The examples provided in figure 12 are explicitly designed to highlight the fragility of using traditional machine learning metrics to evaluate the out-of-sample practicability of predictions in the context of LOB forecasting. In particular, they constitute two ‘extreme’ scenarios where traditional machine learning metrics take values far from the ones given by $p_{T}$, remarking the potential distance of academically acceptable findings and actually practicable ones. Indeed, in this application domain, we are more interested in the chronological location of prediction errors rather than in the number of their occurrence. To be more specific, we are interested in (i) having at least one correct prediction in correspondence of each ‘Down’ or ‘Up’ movement; and, consequently, (ii) in not having any premature closing signal for an existing open position. The nature and the number of other errors are tolerable when these two conditions are satisfied. In real-world scenarios, also probabilities associated with forecasts should be taken into account. Indeed, we can decide to enter or exit a position based on the probability associated with the forecast (i.e. the strength of the signal).

**Figure 13.**
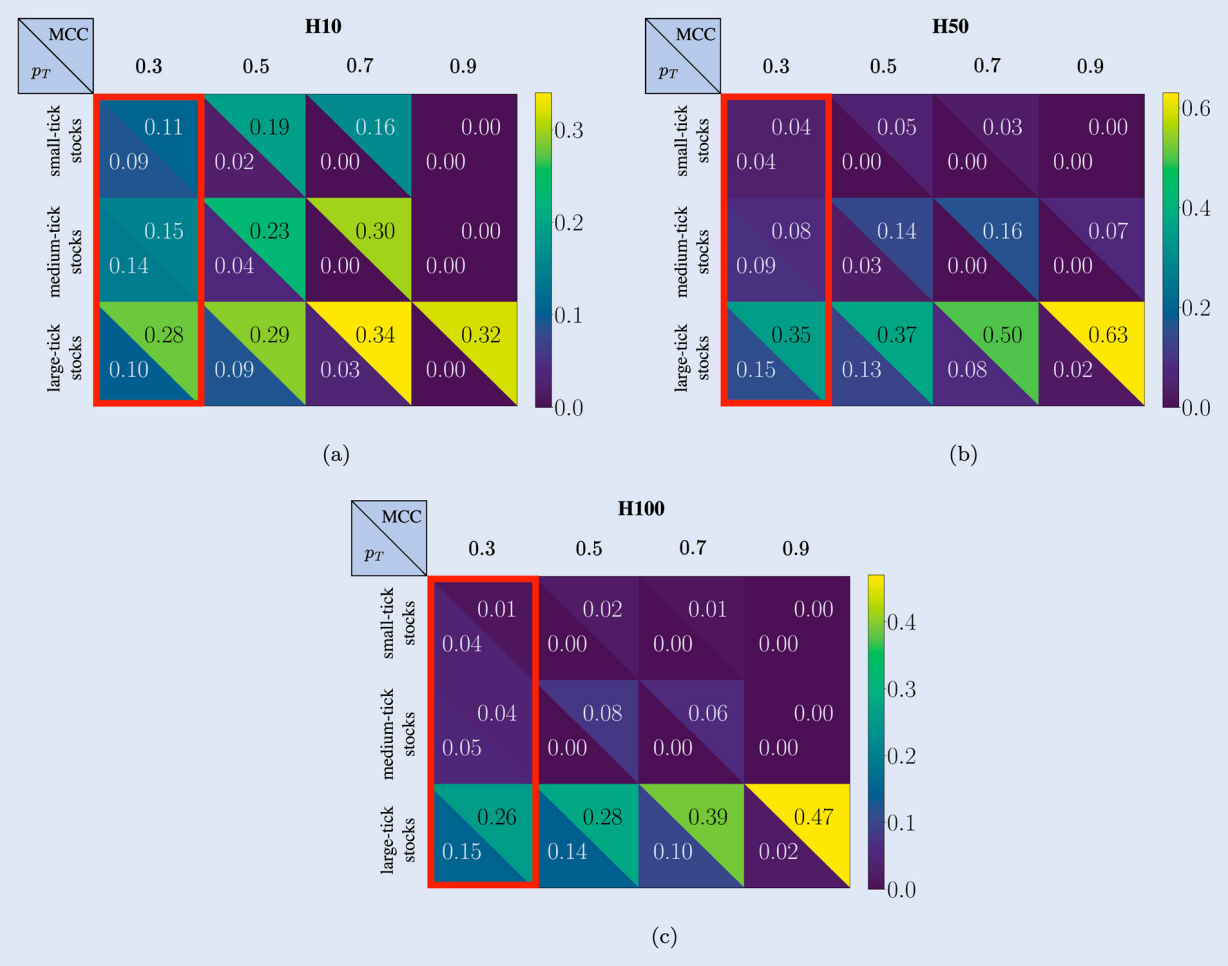
Coarse-grained representation of the behavior of the average $p_{T}$ and MCC at HΔτ∈{10,50,100}. For each class of stocks, we compute the average value for both metrics applying different probability thresholds (i.e. 0.3, 0.5, 0.7, 0.9). We notice two different behaviors that remain consistent across different scenarios: (i) $p_{T}$ decreases for increasing probability thresholds and increases moving from small-tick stocks to large-tick stocks; (ii) the MCC increases for increasing probability thresholds (this is more evident moving to longer prediction horizons) and increases also moving from small-tick stocks to large-tick stocks. We highlight in red the scenarios where no probability threshold is applied (i.e. the signal's sequence is untouched).

These metrics are studied at different granularity levels in figure 13 (i.e. coarse-grained representation) and in tables 8, 9, 10 (i.e. fine-grained representation). Specifically, in figure 13, for each class of stocks, we compute the average value for $p_{T}$ and MCC applying different probability thresholds (0.3, 0.5, 0.7, 0.9) and we notice two different behaviors that remain consistent across different scenarios: (i) $p_{T}$ decreases for increasing probability thresholds and increases moving from small-tick stocks to large-tick stocks; (ii) the MCC increases for increasing probability thresholds (this is more evident moving to longer prediction horizons) and increases also moving from small-tick stocks to large-tick stocks. On one side these findings highlight the relevance of the positioning of the signal. By applying different probability thresholds we may break the signal's sequence, and even if the performance of classical machine learning metrics increases because of the increase of the strength of the signal, we are not able to correctly manage positions. On the other hand they highlight, one more time, the impact of the microstructural properties of the stocks on the signal's usability: overall large-tick stocks demonstrate to offer higher probabilities to actually operate trading in a fully automated way compared to small-tick stocks).

**Table 8.** Strategy-oriented, assumption-free study on the practicability of deep learning forecasts. For HΔτ∈{10}, we report the stock's PT, $p_{T}$, MCC and F1 score with the application of a probability threshold equal to 0.3 (i.e. no threshold), 0.5, 0.7 and 0.9. Being $p_{T}$ a computational expensive metric, results reported in this Table refers to the first 50% of available data only.

H10													
		0.3			0.5			0.7			0.9		
Ticker	PT	$p_{T}$	MCC	F1	$p_{T}$	MCC	F1	$p_{T}$	MCC	F1	$p_{T}$	MCC	F1
CHTR	84 751	0.06	0.09	0.37	0.00	0.13	0.37	0.00	0.04	0.29	0.00	0.00	0.00
GOOG	297 533	0.04	0.20	0.46	0.00	0.33	0.53	0.00	0.28	0.39	0.00	0.00	0.00
GS	99 858	0.08	0.08	0.30	0.01	0.13	0.33	0.00	0.00	0.22	0.00	0.00	0.00
IBM	131 162	0.14	0.10	0.36	0.04	0.17	0.35	0.00	0.12	0.30	0.00	0.00	0.00
MCD	132 481	0.10	0.12	0.41	0.02	0.20	0.44	0.00	0.29	0.51	0.00	0.00	0.00
NVDA	233 588	0.12	0.08	0.35	0.02	0.16	0.37	0.00	0.23	0.42	0.00	0.00	0.13
AAPL	483 853	0.15	0.17	0.42	0.05	0.23	0.46	0.00	0.33	0.50	0.00	0.00	0.29
ABBV	79 782	0.14	0.15	0.41	0.04	0.25	0.47	0.00	0.23	0.41	0.00	0.02	0.33
PM	80 043	0.13	0.13	0.38	0.04	0.22	0.43	0.00	0.35	0.47	0.00	0.00	0.00
BAC	27 155	0.07	0.30	0.45	0.06	0.30	0.46	0.04	0.36	0.50	0.01	0.40	0.54
CSCO	69 914	0.10	0.31	0.50	0.08	0.32	0.51	0.03	0.37	0.55	0.00	0.33	0.54
KO	37 589	0.11	0.24	0.46	0.09	0.25	0.47	0.04	0.28	0.50	0.00	0.20	0.45
ORCL	62 514	0.12	0.32	0.49	0.11	0.33	0.50	0.04	0.40	0.56	0.00	0.39	0.50
PFE	38 853	0.09	0.28	0.46	0.08	0.29	0.47	0.03	0.36	0.54	0.00	0.36	0.52
VZ	87 383	0.11	0.27	0.48	0.09	0.27	0.49	0.02	0.31	0.52	0.00	0.21	0.44

**Table 9.** Strategy-oriented, assumption-free study on the practicability of deep learning forecasts. For HΔτ∈{50}, we report the stock's PT, $p_{T}$, MCC and F1 score with the application of a probability threshold equal to 0.3 (i.e. no threshold), 0.5, 0.7 and 0.9. Being $p_{T}$ a computational expensive metric, results reported in this Table refers to the first 50% of available data only.

H50													
		0.3			0.5			0.7			0.9		
Ticker	PT	$p_{T}$	MCC	F1	$p_{T}$	MCC	F1	$p_{T}$	MCC	F1	$p_{T}$	MCC	F1
CHTR	54 303	0.03	0.05	0.35	0.01	0.06	0.34	0.00	0.09	0.21	0.00	0.00	0.06
GOOG	196 149	0.06	0.09	0.36	0.01	0.13	0.36	0.00	−0.03	0.26	0.00	0.00	0.00
GS	55 020	0.05	0.02	0.29	0.00	0.02	0.22	0.00	0.02	0.13	0.00	0.00	0.02
IBM	64 859	0.06	0.03	0.28	0.01	0.02	0.20	0.00	0.00	0.22	0.00	0.00	0.00
MCD	73 427	0.04	0.04	0.31	0.01	0.07	0.32	0.00	0.07	0.36	0.00	0.00	0.00
NVDA	104 414	0.01	0.01	0.19	0.00	0.00	0.12	0.00	0.00	0.14	0.00	0.00	0.00
AAPL	218 318	0.08	0.10	0.34	0.04	0.18	0.45	0.00	0.16	0.47	0.00	0.00	0.00
ABBV	38 321	0.08	0.08	0.28	0.03	0.12	0.27	0.00	0.23	0.48	0.00	0.21	0.54
PM	42 523	0.10	0.07	0.37	0.01	0.12	0.34	0.00	0.10	0.31	0.00	0.00	0.00
BAC	20 711	0.11	0.43	0.61	0.10	0.44	0.62	0.07	0.57	0.71	0.02	0.69	0.76
CSCO	48 673	0.17	0.36	0.57	0.15	0.38	0.58	0.12	0.49	0.65	0.06	0.60	0.68
KO	25 581	0.16	0.33	0.54	0.15	0.34	0.55	0.10	0.45	0.63	0.03	0.64	0.75
ORCL	41 516	0.16	0.34	0.55	0.15	0.37	0.57	0.08	0.51	0.67	0.00	0.65	0.71
PFE	25 519	0.15	0.38	0.57	0.14	0.40	0.59	0.08	0.59	0.72	0.00	0.74	0.79
VZ	51 748	0.12	0.25	0.48	0.11	0.29	0.50	0.05	0.38	0.56	0.00	0.47	0.61

**Table 10.** Strategy-oriented, assumption-free study on the practicability of deep learning forecasts. For HΔτ∈{100}, we report the stock's PT, $p_{T}$, MCC and F1 score with the application of a probability threshold equal to 0.3 (i.e. no threshold), 0.5, 0.7 and 0.9. Being $p_{T}$ a computational expensive metric, results reported in this Table refers to the first 50% of available data only.

H100													
		0.3			0.5			0.7			0.9		
Ticker	PT	$p_{T}$	MCC	F1	$p_{T}$	MCC	F1	$p_{T}$	MCC	F1	$p_{T}$	MCC	F1
CHTR	42 794	0.04	0.03	0.34	0.00	0.03	0.23	0.00	0.03	0.08	0.00	0.00	0.16
GOOG	15 0428	0.06	0.02	0.32	0.00	0.01	0.21	0.00	-0.00	0.19	0.00	0.00	0.01
GS	39 687	0.06	0.01	0.29	0.00	0.00	0.04	0.00	0.00	0.03	0.00	0.00	0.00
IBM	45 758	0.03	−0.01	0.27	0.00	0.01	0.21	0.00	0.00	0.06	0.00	0.00	0.00
MCD	52 440	0.04	0.01	0.28	0.00	0.04	0.21	0.00	0.00	0.02	0.00	0.00	0.00
NVDA	70 535	0.03	0.00	0.31	0.00	0.01	0.07	0.00	0.00	0.00	0.00	0.00	0.00
AAPL	153 620	0.06	0.06	0.32	0.00	0.17	0.46	0.00	0.00	0.30	0.00	0.00	0.00
ABBV	26 504	0.05	0.04	0.30	0.01	0.06	0.20	0.00	0.18	0.29	0.00	0.00	0.00
PM	30 333	0.05	0.01	0.30	0.00	0.01	0.16	0.00	0.00	0.05	0.00	0.00	0.00
BAC	15 693	0.19	0.35	0.55	0.19	0.36	0.55	0.16	0.46	0.60	0.09	0.62	0.70
CSCO	39 307	0.16	0.30	0.53	0.15	0.33	0.56	0.13	0.44	0.61	0.02	0.56	0.65
KO	19 119	0.13	0.25	0.50	0.12	0.27	0.51	0.08	0.37	0.57	0.02	0.52	0.62
ORCL	31 524	0.17	0.25	0.48	0.16	0.30	0.51	0.11	0.43	0.55	0.00	0.25	0.49
PFE	19 621	0.13	0.28	0.50	0.12	0.31	0.52	0.08	0.42	0.59	0.01	0.55	0.58
VZ	37 859	0.09	0.11	0.31	0.08	0.13	0.31	0.03	0.20	0.35	0.00	0.31	0.40

Deepening the analysis at the the level of specific stocks, looking at tables 8, 9, 10, we notice that, for the class of *small-tick stocks*, at H10, the average PT is $1.63\times 10^{5}$. Without the application of any threshold, looking at $p_{T}$, we observe a separation between stocks: the first set is made of CHTR, GOOG and GS and is characterized by an average $p_{T}$ equal to 0.06, while the second one is made of IBM, MCD and NVDA and is characterized by an average $p_{T}$ equal to 0.12. Such a separation, which was not evident in figure 10, is directly linked to the microstructural properties of the considered stocks. Indeed, as observed in figures 4 and 6, assets belonging to the second family present less extreme realizations of the spread and of the actual LOB's depth, making them structurally more similar to large-tick stocks and more suitable to be treated as input for a deep learning model.^6^ We remark that the above-mentioned statistical properties of the LOB can be effectively exploited by the deep learning model thanks to the rough balancing in class distribution observed at H10 (see table 5). As we point out later in this Section, moving to HΔτ∈{50,100}, this effect will vanish due to a stronger class imbalance. For all the small-tick stocks, the decrease of $p_{T}$ is fast when applying probability thresholds. Specifically, net of minor oscillations, the probability of correctly executing a trade at a threshold larger than 0.5 is zero. For *medium-tick stocks*, the average PT (i.e. $2.1\times 10^{5}$) is strongly biased by the behavior of AAPL. In contrast, $p_{T}$ (which has an average value equal to 0.14) has similar realizations for all the stocks. Also in this case, the decrease in $p_{T}$ is fast when thresholds are applied, and the probability of correctly executing a transaction at a threshold larger than 0.5 is 0. The behavior is different when we analyze *large-tick stocks*. In this case, the average PT equals $5.3\times 10^{4}$, which is almost 1/3 of the one detected for small-tick stocks. Even if the number of LOB updates is much higher for large-tick stocks than for small-tick stocks, the number of mid-price changes, and, consequently, the number of potentially exploitable transactions, follows an inverse pattern, being, on average, one order of magnitude higher for small-tick stocks than for large-tick stocks. Also the application of probability thresholds in large-tick stocks leads to different results. Indeed, without applying any threshold, the average $p_{T}$ value for this class of stocks equals 0.10, with a smoother decrease for higher threshold values. In this case, the probability of correctly executing a transaction is ≈0 only for a threshold equal to 0.9. To deepen the $p_{T}$-related results discussed for H10, it is useful to exploit the average confusion matrices in figure 7 as an instrument to understand the distribution of forecasting errors. In this context, indeed, we observe that the non-negligible frequency of reciprocal misclassifications between the extreme classes (−1 and 1) for models trained on small- and medium-tick stocks, directly determines a sub-optimal management of the opening/closing of existing or new positions. Conversely, for large-tick stocks, errors' concentration towards the misclassification of the two extreme classes as 0 guarantees a reduced impact on the management of the opening/closing of existing or new positions. Moving to H50, for small-tick stocks, we observe a decrease in the average PT, which is equal to $9.1\times 10^{4}$. Also the $p_{T}$, for all the probability thresholds, is consistently lower than the one observed at H10. Indeed, without the application of any threshold (i.e. 0.3), the average $p_{T}$ is equal to 0.04, while the probability of correctly executing a transaction at a threshold larger than 0.5 is always 0. Differently from what is observed at H10, stocks have no intra-class separation. These findings are also true for medium-tick stocks. In this case, the average PT is equal to $9.9\times 10^{4}$, while the average $p_{T}$ is equal to 0.09. One more time, a symmetrically different trend is observed for large-tick stocks. In this case, it remains true that the average number of potentially executable transactions (i.e. $3.6\times 10^{4}$) decreases when compared to the one observed at H10, however, curiously, the average $p_{T}$ increases reaching a value of 0.15. The analysis of confusion matrices (see figure 8) reveals that, for small- and medium-tick stocks, the lowest realizations of $p_{T}$ compared to H10, are directly linked to a more evident tendency to mix the extreme classes (−1 and 1) which directly determine the opening/closing of existing or new positions. Conversely, for large-tick stocks, a decrease of these types of errors in favor of a misclassification of extreme classes toward the central one (i.e. class 0), determines an increase in realizations of $p_{T}$ compared to H10. Similar findings can be detected moving to H100. In this case, for small-tick stocks, we observe a further decrease in the average PT, which is equal to $6.7\times 10^{4}$. Without applying any threshold, the average $p_{T}$ is in line with the one observed at H50, while the probability of correctly executing a transaction with the application of a threshold larger than 0.3 is always 0. Also in this case, there is no intra-class separation among stocks. For medium-tick stocks, the average PT is equal to $7.0\times 10^{4}$, while the average $p_{T}$ is lower than the one observed at H50, with a value equal to 0.05. One more time, for large-tick stocks, we observe that even if it remains true that the average number of potentially executable transactions (i.e. $2.7\times 10^{4}$) decreases when compared to the one observed at H50, the average $p_{T}$ remains unchanged with a value equal to 0.15. In this case, the probability of correctly executing a transaction is remarkably higher than 0 for probability thresholds ≤0.7. In this case, results of the analysis of confusion matrices (see figure 9) are identical to the ones performed at H50.

To conclude the analysis of the results, we draw the attention to the uniqueness of the pattern of $p_{T}$ observed for large-tick stocks across the different horizons. Microstructural properties alone cannot fully explain this behavior. Instead, we must also consider the primary role of class distributions at HΔτ∈{10,50,100} in determining this trend. As we have previously noted in tables 3, 4 and 5, class imbalances follow two symmetrically different patterns for small- and large-tick stocks. The first class of assets has a more balanced distribution at H10, while the second class of assets achieves a stable balance at HΔτ∈{50,100}. Overall, this result, combined with the balanced sampling technique used during the training stage, as well as the aggregate statistical properties of the LOB for different classes of stocks, sheds lights on the practicability of forecasts and issues related to the use of deep learning forecasting techniques on LOB data.

## 8. Conclusion and future work

Understanding and forecasting Limit Order Book (LOB) dynamics is a fundamental challenge in financial markets. LOBs are complex systems where price formation is influenced by microstructural properties, high-frequency trading activity, and order flow dynamics. Accurate forecasting is particularly difficult due to the stochastic nature of financial markets, the low signal-to-noise ratio, and structural variations across different stocks. While significant research has been conducted on LOB modeling and predictive analytics, a standardized framework for integrating microstructural analysis with forecasting has remained largely absent.

To address this gap, we develop LOBFrame, an open-source framework designed to facilitate the systematic study and evaluation of LOB forecasting models. Our approach is twofold: (i) we analyze the microstructural characteristics of a diverse set of 15 highly liquid NASDAQ stocks over a three-year period (2017–2019), categorizing them based on their tick size, and (ii) we leverage these insights to enhance and understand limitations of forecasting methodologies. We also establish clear quantitative benchmarks that enable us to differentiate between small-tick, medium-tick, and large-tick stocks.

**On the microstructural side,** we analyze various LOB properties to understand how different tick sizes influence market behavior. While new measures such as the information richness ratio (Kolm *et al.* 2023) have been proposed, we find that many observed behavioral clusters can be directly attributed to tick-size-driven effects.

**On the forecasting side**, LOBFrame provides a scalable and modular system for processing large-scale LOB data, integrating cutting-edge deep learning methodologies. It offers a standardized pipeline for data transformation, training, validation, and trading simulation. This allows for rigorous model evaluation and ensures comparability across different forecasting approaches. In this study, we build upon DeepLOB, a state-of-the-art deep learning model for LOB forecasting, and propose an enhanced labeling procedure that improves usability in high-frequency trading strategies. Additionally, we introduce a data-parsimonious pipeline to address inherent class imbalances in LOB datasets.

To assess forecasting performance, we measure the Matthews Correlation Coefficient (MCC) across three prediction horizons (expressed in LOB updates) at varying confidence levels (i.e. probability thresholds). Our findings reveal a strong correlation between tick size and forecast accuracy:

- Large-tick stocks exhibit the highest predictability, with robust performance across different horizons.
- Small-tick stocks present a weaker predictive signal, requiring more sophisticated modeling approaches.

However, we also highlight a critical practical limitation: the usefulness of this predictive signal depends significantly on the availability of low-latency hardware infrastructure. Even though deep learning models can detect market inefficiencies, their real-world utility is constrained by execution delays inherent to trading systems.

Finally, going deeper with the study the practicability of obtained forecasts in real-world scenarios, we develop a strategy-oriented, assumptions-free and class imbalances-immune methodology to compute the probability of executing a correct transaction using the forecasts of the chosen model. We argue that this approach is more general than the one based on estimating the PnL of a single strategy on historical data, which is often based on unrealistic assumptions. We show that assessing the probability of executing a correct transaction is a more robust procedure compared to those used in traditional deep learning, as it correctly takes into account the impact of the chronological location of errors on the performance.

Our paper provides a robust methodology and a data pipeline that bridges the analysis and modeling of microstructural properties of LOB data with the forecast of LOB dynamics, providing specific indications to practitioners on the stocks characteristics and factors driving the forecast performance. Indeed, there are a number of research avenues yet to be explored. Specifically, a cross-exchange validation of our results is needed. In addition, we remark the need for structured testing of different deep learning models on heterogeneous classes of stocks. This analysis would aim to unveil how the models' architectural peculiarities can be exploited to handle specific challenges coming from, for example, the sparser LOB structure characterizing small- to medium-tick stocks. This includes further studies on the potentialities of transformer models (Vaswani *et al.* 2017, Zhou *et al.* 2021, Wen *et al.* 2022, Zeng *et al.* 2023), diffusion models (Sohl-Dickstein *et al.* 2015, Song and Ermon 2019, Ho *et al.* 2020, Nichol and Dhariwal 2021) and graph-based models (Wang and Aste 2022, Briola and Aste 2023, Briola *et al.* 2023b, Wang *et al.* 2023) in the application domain considered in this paper.

## Data Availability

The data that support the findings of this study are available from LOBSTER data provider (LOBSTER 2023). Restrictions apply to the availability of these data, which were used under license for this study. The code is available at https://github.com/FinancialComputingUCL/LOBFrame.

## Footnotes

1 https://github.com/FinancialComputingUCL/LOBFrame.

2 The value of *θ* varies across exchanges and, within the same exchange, for a single stock, it can vary across time as a function of the price attained by the asset.

3 The reader should be aware that, in the Python code related to the current research paper, the labeling schema is: 0→Down, 1→Stable, 2→Up. In equation (2) we use labels −1, 0 and 1, respectively, to enhance the paper's readability and remain coherent with the standard used in the related literature (Zhang *et al.* 2019).

4 We remark that results of trading simulations are not part of this work because of the reasons reported in section 7.2.

5 We stress that theoretical findings discussed in section 7 are independent of the choice of the model. Indeed, in this work, we decide to use DeepLOB only because it fully respects the following three criteria: (i) availability of the original code used in the experiments; (ii) data-driven design of the model's architecture; (iii) community's recognition as a state-of-the-art model in the field.

6 These findings are also coherent with the ones observed in table A1, where for stocks belonging to the second family, we observe an average (computed across years) IR value equal to 1.85, which is higher than the one observed for the stocks belonging to the first family of stocks (i.e. 1.71).

## Appendix 1. Information richness ratio

An example of a derived microstructural property is the stocks' ‘information richness’ (IR) ratio. This measure was introduced by Kolm *et al.* (2023), and is defined as the logarithm of the ratio of the total number of LOB's updates to price changes (i.e. events occurring at the best levels of the LOB).

As one can notice from table A1, where we report for for each stock (belonging to one of the three tick size classes) the total number of LOB updates, the total number of price changes and the associated ‘information-richness’ (IR) ratio, an evident clustering behavior is detectable based on the stocks' tick-size.

**Table A1.** In this Table, for each stock, we report the total number of LOB updates, the total number of price changes and the associated ‘information-richness’ (IR) ratio.

	2017			2018			2019		
	LOB	Price	IR	LOB	Price	IR	LOB	Price	IR
Ticker	Updates	Changes		Updates	Changes		Updates	Changes	
CHTR	1.25e+07	2.89e+06	1.46	1.84e+07	4.12e+06	1.49	1.52e+07	3.84e+06	1.38
GOOG	3.39e+07	5.23e+06	1.87	1.37e+08	1.47e+07	2.24	1.10e+08	1.27e+07	2.16
GS	1.84e+07	3.97e+06	1.54	3.55e+07	6.69e+06	1.67	2.79e+07	5.50e+06	1.62
IBM	2.35e+07	2.84e+06	2.11	4.10e+07	6.17e+06	1.89	4.18e+07	5.13e+06	2.10
MCD	2.44e+07	2.70e+06	2.20	3.48e+07	6.09e+06	1.74	2.69e+07	4.19e+06	1.86
NVDA	7.31e+07	1.26e+07	1.76	9.05e+07	2.16e+07	1.43	8.37e+07	1.62e+07	1.64
AAPL	1.91e+08	1.25e+07	2.72	2.22e+08	2.88e+07	2.04	2.30e+08	2.47e+07	2.23
ABBV	3.53e+07	3.38e+06	2.35	3.40e+07	5.85e+06	1.76	5.03e+07	4.54e+06	2.40
PM	3.40e+07	3.66e+06	2.23	3.88e+07	4.48e+06	2.16	3.96e+07	3.48e+06	2.43
BAC	9.58e+07	5.91e+05	5.09	1.62e+08	1.26e+06	4.85	1.23e+08	7.06e+05	5.16
CSCO	6.76e+07	4.29e+05	5.06	1.69e+08	2.59e+06	4.18	1.42e+08	1.80e+06	4.37
KO	3.71e+07	4.59e+05	4.39	6.93e+07	1.53e+06	3.82	7.27e+07	1.13e+06	4.17
ORCL	4.80e+07	9.23e+05	3.95	1.11e+08	2.86e+06	3.66	1.03e+08	1.96e+06	3.96
PFE	4.40e+07	5.13e+05	4.45	9.68e+07	1.84e+06	3.96	9.70e+07	1.18e+06	4.41
VZ	4.84e+07	1.18e+06	3.71	9.16e+07	3.19e+06	3.36	9.24e+07	1.81e+06	3.93

As expected from results of analyses in section 6, small-tick stocks, inherently characterized by a lower number of updates, are exposed to a less granular and information-poor price discovery. In this case, the maximum IR value (i.e. 2.24) is reached by GOOG during 2018, while the minimum IR value (i.e. 1.38) is reached by CHTR in 2019. Medium-tick stocks are instead characterized by higher IR values with a maximum value of 2.72 achieved by AAPL in 2017 and a minimum value of 1.76 for ABBV in 2018. It is worth noting that, differently from previous analyses, a clear separation between AAPL and the other two stocks is not evident. Lastly, large-tick stocks are characterized by higher values of IR and, consequently, by a more granular and information-rich price discovery. The maximum realization (i.e. 5.16) is detected for BAC during 2017, while the minimum realization (i.e. 3.36) is detected for VZ during 2018.

The analysis of the stocks' ‘information richness’ needs further discussion. Indeed, in the original paper by Kolm *et al.* (2023), the authors claim it is a measure for stocks' predictability; this is only partially true. As we empirically show here, there is a direct mapping between the ‘information-richness’ of a stock and its tick-size; consequently, the tick-size itself could be used as a proxy measure of a stock's predictability.

## Appendix 2. Statistical significance of traditional machine learning metrics

**Table A2.** DeepLOB model's year-wise Matthews Correlation Coefficient (MCC) at H10 for different confidence levels (i.e. probability thresholds). Statistical significance obtained through a parametric *t*-test is represented through asterisks. *p*-values >0.05 are not marked. *p*-values <0.001 are marked as ${\;}^{***}$. 0.001≤ *p*-values <0.01 are marked as ${\;}^{**}$. 0.01≤ *p*-values <0.05 are marked as ${\;}^{*}$. The ‘/’ symbol is used when applying a probability thresholds leads to the absence of any remaining forecast.

		H10						
Ticker	Year	0.3	0.4	0.5	0.6	0.7	0.8	0.9
CHTR	2017	$7.60e-02^{***}$	$7.97e-02^{***}$	$8.75e-02^{***}$	$9.32e-02^{***}$	−1.88e−02	/	/
	2018	$1.07e-01^{***}$	$1.13e-01^{***}$	$2.13e-01^{***}$	$1.48e-01^{***}$	0.00e+00	0.00e+00	/
	2019	$1.05e-01^{***}$	$1.26e-01^{***}$	$1.65e-01^{***}$	$2.30e-01^{***}$	$1.73e-01^{***}$	/	/
GOOG	2017	$1.40e-01^{***}$	$1.62e-01^{***}$	$2.46e-01^{***}$	$3.30e-01^{***}$	$5.53e-01^{***}$	/	/
	2018	$2.42e-01^{***}$	$2.66e-01^{***}$	$3.43e-01^{***}$	$2.52e-01^{***}$	$9.73e-02^{***}$	0.00e+00	/
	2019	$2.01e-01^{***}$	$2.28e-01^{***}$	$3.06e-01^{***}$	$3.69e-01^{***}$	$1.05e-01^{***}$	0.00e+00	/
GS	2017	$1.87e-01^{***}$	$2.13e-01^{***}$	$3.17e-01^{***}$	$3.52e-01^{***}$	1.24e−02	0.00e+00	/
	2018	$5.80e-02^{***}$	$6.25e-02^{***}$	$1.31e-01^{***}$	$2.22e-01^{***}$	/	/	/
	2019	$5.13e-02^{***}$	$4.89e-02^{***}$	$8.72e-03^{***}$	4.34e−03	0.00e+00	/	/
IBM	2017	$1.17e-01^{***}$	$1.38e-01^{***}$	$1.81e-01^{***}$	$3.51e-02^{**}$	0.00e+00	/	/
	2018	$9.31e-02^{***}$	$9.72e-02^{***}$	$1.36e-01^{***}$	$1.97e-01^{***}$	$2.53e-01^{***}$	$2.61e-01^{***}$	/
	2019	$1.25e-01^{***}$	$1.64e-01^{***}$	$2.39e-01^{***}$	$1.96e-01^{***}$	$7.88e-02^{***}$	−3.32e−02	/
MCD	2017	$1.98e-01^{***}$	$2.16e-01^{***}$	$2.97e-01^{***}$	$4.12e-01^{***}$	$4.74e-01^{***}$	$5.85e01^{***}$	/
	2018	$7.05e-02^{***}$	$9.29e-02^{***}$	$1.60e-01^{***}$	$1.30e-01^{***}$	$1.18e-01^{**}$	/	/
	2019	$8.84e-02^{***}$	$1.04e-01^{***}$	$1.54e-01^{***}$	$1.27e-01^{**}$	/	/	/
NVDA	2017	$2.40e-02^{***}$	$2.34e-02^{***}$	$2.95e-02^{***}$	$3.05e-02^{***}$	$1.54e-01^{***}$	6.14e−02	/
	2018	$1.16e-01^{***}$	$1.20e-01^{***}$	$2.33e-01^{***}$	$3.98e-01^{***}$	$3.83e-01^{***}$	/	/
	2019	$1.08e-01^{***}$	$1.36e-01^{***}$	$2.18e-01^{***}$	$2.86e-01^{***}$	$2.44e-01^{***}$	$1.73e-01^{***}$	0.00e+00
AAPL	2017	$2.80e-01^{***}$	$2.88e-01^{***}$	$3.30e-01^{***}$	$4.10e-01^{***}$	$5.07e-01^{***}$	$6.14e-01^{***}$	0.00e+00
	2018	$5.92e-02^{***}$	$7.34e-02^{***}$	$1.35e-01^{***}$	$2.20e-01^{***}$	$1.59e-01^{***}$	1.75e−02	0.00e+00
	2019	$1.82e-01^{***}$	$1.93e-01^{***}$	$2.59e-01^{***}$	$3.28e-01^{***}$	$3.87e-01^{***}$	$1.41e-01^{***}$	0.00e+00
ABBV	2017	$1.66e-01^{***}$	$1.74e-01^{***}$	$2.66e-01^{***}$	$3.11e-01^{***}$	$3.40e-01^{***}$	$3.64e-01^{***}$	$6.90e-02^{***}$
	2018	$8.02e-02^{***}$	$9.28e-02^{***}$	$1.84e-01^{***}$	$2.12e-01^{***}$	$1.56e-01^{**}$	/	/
	2019	$1.34e-01^{***}$	$1.47e-01^{***}$	$2.16e-01^{***}$	$2.94e-01^{***}$	$3.48e-01^{***}$	$2.40e-01^{***}$	/
PM	2017	$1.60e-01^{***}$	$1.78e-01^{***}$	$2.61e-01^{***}$	$3.71e-01^{***}$	$4.34e-01^{***}$	$4.80e-01^{***}$	/
	2018	$7.73e-02^{***}$	$8.37e-02^{***}$	$1.10e-01^{***}$	$1.14e-01^{***}$	$9.59e-02^{***}$	$3.90e-01^{**}$	/
	2019	$1.19e-01^{***}$	$1.27e-01^{***}$	$1.65e-01^{***}$	$2.18e-01^{***}$	$2.61e-01^{***}$	$3.57e-01^{***}$	/
BAC	2017	$2.85e-01^{***}$	$2.86e-01^{***}$	$2.93e-01^{***}$	$3.30e-01^{***}$	$3.78e-01^{***}$	$4.38e-01^{***}$	$4.53e-01^{***}$
	2018	$3.33e-01^{***}$	$3.33e-01^{***}$	$3.37e-01^{***}$	$3.65e-01^{***}$	$3.93e-01^{***}$	$4.26e-01^{***}$	$4.65e-01^{***}$
	2019	$2.86e-01^{***}$	$2.86e-01^{***}$	$2.88e-01^{***}$	$3.17e-01^{***}$	$3.46e-01^{***}$	$3.64e-01^{***}$	$3.48e-01^{***}$
CSCO	2017	$2.51e-01^{***}$	$2.51e-01^{***}$	$2.52e-01^{***}$	$2.78e-01^{***}$	$2.92e-01^{***}$	$2.90e-01^{***}$	$2.83e-01^{***}$
	2018	$2.96e-01^{***}$	$2.97e-01^{***}$	$3.04e-01^{***}$	$3.32e-01^{***}$	$3.61e-01^{***}$	$3.96e-01^{***}$	$4.54e-01^{***}$
	2019	$3.52e-01^{***}$	$3.54e-01^{***}$	$3.66e-01^{***}$	$4.07e-01^{***}$	$4.47e-01^{***}$	$4.79e-01^{***}$	$3.60e-01^{***}$
KO	2017	$2.37e-01^{***}$	$2.37e-01^{***}$	$2.42e-01^{***}$	$2.68e-01^{***}$	$2.98e-01^{***}$	$3.42e-01^{***}$	$3.83e-01^{***}$
	2018	$2.93e-01^{***}$	$2.94e-01^{***}$	$2.99e-01^{***}$	$3.16e-01^{***}$	$3.29e-01^{***}$	$3.28e-01^{***}$	$2.55e-01^{***}$
	2019	$3.10e-01^{***}$	$3.11e-01^{***}$	$3.20e-01^{***}$	$3.55e-01^{***}$	$3.86e-01^{***}$	$4.04e-01^{***}$	$1.73e-01^{***}$
ORCL	2017	$3.18e-01^{***}$	$3.19e-01^{***}$	$3.23e-01^{***}$	$3.72e-01^{***}$	$4.29e-01^{***}$	$4.74e-01^{***}$	$4.73e-01^{***}$
	2018	$3.23e-01^{***}$	$3.24e-01^{***}$	$3.30e-01^{***}$	$3.58e-01^{***}$	$3.72e-01^{***}$	$3.32e-01^{***}$	$1.18e-01^{***}$
	2019	$3.10e-01^{***}$	$3.11e-01^{***}$	$3.20e-01^{***}$	$3.49e-01^{***}$	$3.79e-01^{***}$	$4.23e-01^{***}$	$5.15e-01^{***}$
PFE	2017	$2.52e-01^{***}$	$2.55e-01^{***}$	$2.67e-01^{***}$	$3.10e-01^{***}$	$3.69e-01^{***}$	$4.09e-01^{***}$	$3.29e-01^{***}$
	2018	$2.72e-01^{***}$	$2.73e-01^{***}$	$2.80e-01^{***}$	$2.99e-01^{***}$	$3.13e-01^{***}$	$3.07e-01^{***}$	$1.53e-01^{***}$
	2019	$2.86e-01^{***}$	$2.87e-01^{***}$	$2.92e-01^{***}$	$3.21e-01^{***}$	$3.53e-01^{***}$	$3.96e-01^{***}$	$4.32e-01^{***}$
VZ	2017	$3.13e-01^{***}$	$3.16e-01^{***}$	$3.23e-01^{***}$	$3.63e-01^{***}$	$4.08e-01^{***}$	$4.52e-01^{***}$	$3.85e-01^{***}$
	2018	$2.42e-01^{***}$	$2.45e-01^{***}$	$2.61e-01^{***}$	$2.88e-01^{***}$	$3.06e-01^{***}$	$2.97e-01^{***}$	$1.81e-01^{***}$
	2019	$2.86e-01^{***}$	$2.87e-01^{***}$	$2.88e-01^{***}$	$2.99e-01^{***}$	$2.97e-01^{***}$	$2.80e-01^{***}$	$1.92e-01^{***}$

**Table A3.** DeepLOB model's year-wise Matthews Correlation Coefficient (MCC) at H50 for different confidence levels (i.e. probability thresholds). Statistical significance obtained through a parametric t-test is represented through asterisks. *p*-values >0.05 are not marked. *p*-values <0.001 are marked as ${\;}^{***}$. 0.001≤ *p*-values <0.01 are marked as ${\;}^{**}$. 0.01≤ *p*-values <0.05 are marked as ${\;}^{*}$. The ‘/’ symbol is used when the application of a probability thresholds implies the absence of any remaining forecast.

		H50						
Ticker	Year	0.3	0.4	0.5	0.6	0.7	0.8	0.9
CHTR	2017	$1.15e-01^{***}$	$1.22e-01^{***}$	$1.40e-01^{***}$	$1.56e-01^{***}$	$1.32e-01^{***}$	0.00e+00	0.00e+00
	2018	$4.04e-02^{***}$	$4.42e-02^{***}$	$6.39e-02^{***}$	$9.08e-02^{***}$	$5.37e-02^{***}$	0.00e+00	0.00e+00
	2019	$1.69e-02^{***}$	$1.90e-02^{***}$	$2.87e-02^{***}$	0.00e+00	0.00e+00	0.00e+00	0.00e+00
GOOG	2017	$2.02e-02^{***}$	$2.36e-02^{***}$	$4.45e-02^{***}$	$6.33e-02^{***}$	$4.95e-02^{***}$	3.74e−02	0.00e+00
	2018	$1.34e-01^{***}$	$1.44e-01^{***}$	$1.48e-01^{***}$	$1.86e-01^{***}$	0.00e+00	/	/
	2019	$1.16e-01^{***}$	$1.32e-01^{***}$	$2.15e-01^{***}$	0.00e+00	/	/	/
GS	2017	$6.44e-02^{***}$	$7.38e-02^{***}$	$8.36e-02^{***}$	$3.82e-02^{***}$	$5.81e-02^{***}$	0.00e+00	0.00e+00
	2018	$1.80e-02^{***}$	$2.72e-02^{***}$	0.00e+00	0.00e+00	0.00e+00	/	/
	2019	−2.80e−04	4.74e−04	−8.48e−04	−2.21e−01	/	/	/
IBM	2017	$3.47e-02^{***}$	$3.50e-02^{***}$	$3.54e-02^{***}$	$3.16e-02^{***}$	0.00e+00	0.00e+00	/
	2018	$4.73e-02^{***}$	$4.71e-02^{***}$	$1.32e-02^{***}$	$2.50e-03^{**}$	1.17e−02	/	/
	2019	$1.72e-02^{***}$	$3.89e-02^{***}$	0.00e+00	/	/	/	/
MCD	2017	$9.19e-02^{***}$	$1.03e-01^{***}$	$1.47e-01^{***}$	$1.57e-01^{***}$	$1.57e-01^{***}$	/	/
	2018	$2.98e-02^{***}$	$3.95e-02^{***}$	$9.07e-02^{***}$	0.00e+00	/	/	/
	2019	$4.90e-03^{***}$	$3.75e-03^{***}$	$3.24e-03^{**}$	4.72e−02	0.00e+00	0.00e+00	/
NVDA	2017	$-1.64e-03^{**}$	−9.20e−04	0.00e+00	0.00e+00	/	/	/
	2018	$1.16e-02^{***}$	$1.87e-02^{***}$	0.00e+00	0.00e+00	0.00e+00	0.00e+00	/
	2019	$9.21e-03^{***}$	1.39e−03	0.00e+00	/	/	/	/
AAPL	2017	$1.97e-01^{***}$	$2.28e-01^{***}$	$3.15e-01^{***}$	$4.16e-01^{***}$	$5.09e-01^{***}$	$6.09e-01^{***}$	/
	2018	$1.05e-02^{***}$	$1.31e-02^{***}$	/	/	/	/	/
	2019	$1.12e-01^{***}$	$1.18e-01^{***}$	$2.34e-01^{***}$	$3.65e-01^{***}$	$7.96e-02^{***}$	0.00e+00	/
ABBV	2017	$1.22e-01^{***}$	$1.24e-01^{***}$	$1.75e-01^{***}$	$3.01e-01^{***}$	$4.69e-01^{***}$	$6.05e-01^{***}$	$7.18e-01^{***}$
	2018	$1.06e-02^{***}$	−2.10e−03	0.00e+00	0.00e+00	/	/	/
	2019	$9.77e-02^{***}$	$1.02e-01^{***}$	$1.89e-01^{***}$	$1.40e-01^{***}$	$1.10e-01^{***}$	0.00e+00	/
PM	2017	$8.66e-02^{***}$	$8.90e-02^{***}$	$1.94e-01^{***}$	$2.57e-01^{***}$	0.00e+00	/	/
	2018	$5.71e-02^{***}$	$6.06e-02^{***}$	$9.23e-02^{***}$	$3.85e-02^{***}$	$3.81e-02^{**}$	/	/
	2019	$7.08e-02^{***}$	$8.18e-02^{***}$	$1.24e-01^{***}$	$7.35e-02^{***}$	/	/	/
BAC	2017	$4.53e-01^{***}$	$4.53e-01^{***}$	$4.59e-01^{***}$	$5.28e-01^{***}$	$6.16e-01^{***}$	$6.98e-01^{***}$	$7.80e-01^{***}$
	2018	$4.57e-01^{***}$	$4.57e-01^{***}$	$4.59e-01^{***}$	$4.94e-01^{***}$	$5.25e-01^{***}$	$5.54e-01^{***}$	$5.90e-01^{***}$
	2019	$4.03e-01^{***}$	$4.05e-01^{***}$	$4.18e-01^{***}$	$4.94e-01^{***}$	$5.81e-01^{***}$	$6.75e-01^{***}$	$7.77e-01^{***}$
CSCO	2017	$3.86e-01^{***}$	$3.86e-01^{***}$	$3.87e-01^{***}$	$4.16e-01^{***}$	$4.53e-01^{***}$	$5.22e-01^{***}$	$6.57e-01^{***}$
	2018	$3.58e-01^{***}$	$3.61e-01^{***}$	$3.85e-01^{***}$	$4.32e-01^{***}$	$4.86e-01^{***}$	$5.52e-01^{***}$	$6.49e-01^{***}$
	2019	$3.69e-01^{***}$	$3.75e-01^{***}$	$4.12e-01^{***}$	$5.00e-01^{***}$	$6.07e-01^{***}$	$6.89e-01^{***}$	$5.83e-01^{***}$
KO	2017	$4.21e-01^{***}$	$4.22e-01^{***}$	$4.34e-01^{***}$	$5.11e-01^{***}$	$5.98e-01^{***}$	$6.94e-01^{***}$	$7.68e-01^{***}$
	2018	$2.94e-01^{***}$	$2.97e-01^{***}$	$3.13e-01^{***}$	$3.52e-01^{***}$	$3.93e-01^{***}$	$4.49e-01^{***}$	$5.60e-01^{***}$
	2019	$3.18e-01^{***}$	$3.21e-01^{***}$	$3.37e-01^{***}$	$3.91e-01^{***}$	$4.58e-01^{***}$	$5.50e-01^{***}$	$6.93e-01^{***}$
ORCL	2017	$4.04e-01^{***}$	$4.07e-01^{***}$	$4.34e-01^{***}$	$5.10e-01^{***}$	$6.00e-01^{***}$	$6.99e-01^{***}$	$7.92e-01^{***}$
	2018	$3.15e-01^{***}$	$3.16e-01^{***}$	$3.29e-01^{***}$	$3.80e-01^{***}$	$4.67e-01^{***}$	$6.04e-01^{***}$	$6.40e-01^{***}$
	2019	$3.17e-01^{***}$	$3.19e-01^{***}$	$3.39e-01^{***}$	$4.02e-01^{***}$	$4.77e-01^{***}$	$5.28e-01^{***}$	$5.10e-01^{***}$
PFE	2017	$4.12e-01^{***}$	$4.13e-01^{***}$	$4.26e-01^{***}$	$5.09e-01^{***}$	$6.21e-01^{***}$	$7.50e-01^{***}$	$8.44e-01^{***}$
	2018	$3.07e-01^{***}$	$3.10e-01^{***}$	$3.40e-01^{***}$	$4.24e-01^{***}$	$5.11e-01^{***}$	$5.82e-01^{***}$	$6.46e-01^{***}$
	2019	$4.13e-01^{***}$	$4.17e-01^{***}$	$4.42e-01^{***}$	$5.38e-01^{***}$	$6.51e-01^{***}$	$7.56e-01^{***}$	$7.33e-01^{***}$
VZ	2017	$3.27e-01^{***}$	$3.33e-01^{***}$	$3.71e-01^{***}$	$4.26e-01^{***}$	$4.78e-01^{***}$	$5.36e-01^{***}$	$6.30e-01^{***}$
	2018	$2.06e-01^{***}$	$2.11e-01^{***}$	$2.46e-01^{***}$	$3.00e-01^{***}$	$3.68e-01^{***}$	$4.76e-01^{***}$	$4.48e-01^{***}$
	2019	$3.07e-01^{***}$	$3.09e-01^{***}$	$3.32e-01^{***}$	$3.90e-01^{***}$	$4.59e-01^{***}$	$5.39e-01^{***}$	$5.83e-01^{***}$

**Table A4.** DeepLOB model's year-wise Matthews Correlation Coefficient (MCC) at H100 for different confidence levels (i.e. probability thresholds). Statistical significance obtained through a parametric t-test is represented through asterisks. *p*-values >0.05 are not marked. *p*-values <0.001 are marked as ${\;}^{***}$. 0.001≤ *p*-values <0.01 are marked as ${\;}^{**}$. 0.01≤ *p*-values <0.05 are marked as ${\;}^{*}$. The ‘/’ symbol is used when the application of a probability thresholds implies the absence of any remaining forecast.

		H100						
Ticker	Year	0.3	0.4	0.5	0.6	0.7	0.8	0.9
CHTR	2017	$4.66e-02^{***}$	$7.13e-02^{***}$	$7.76e-02^{***}$	0.00e+00	0.00e+00	0.00e+00	/
	2018	$3.08e-02^{***}$	$3.25e-02^{***}$	$4.73e-02^{***}$	$7.03e-02^{***}$	$7.49e-02^{***}$	0.00e+00	0.00e+00
	2019	$3.58e-03^{***}$	−1.57e−03	7.23e−03	0.00e+00	0.00e+00	0.00e+00	/
GOOG	2017	$1.25e-02^{***}$	$1.28e-02^{***}$	$8.49e-03^{***}$	3.16e−03	$-5.84e-03^{**}$	$-3.88e-02^{***}$	0.00e+00
	2018	$7.10e-03^{***}$	$6.52e-03^{***}$	0.00e+00	0.00e+00	/	/	/
	2019	$2.39e-02^{***}$	$2.20e-02^{***}$	$1.56e-02^{***}$	0.00e+00	0.00e+00	0.00e+00	/
GS	2017	$1.43e-02^{***}$	$1.42e-02^{***}$	$3.26e-02^{***}$	0.00e+00	0.00e+00	0.00e+00	0.00e+00
	2018	$1.48e-03^{**}$	$8.64e-03^{***}$	0.00e+00	0.00e+00	0.00e+00	0.00e+00	/
	2019	$-6.92e-03^{***}$	$-7.32e-03^{***}$	0.00e+00	0.00e+00	0.00e+00	0.00e+00	/
IBM	2017	$3.78e-03^{***}$	$4.26e-03^{***}$	$1.81e-02^{***}$	0.00e+00	0.00e+00	0.00e+00	0.00e+00
	2018	$4.20e-03^{***}$	$4.35e-02^{***}$	−7.08e−03	0.00e+00	/	/	/
	2019	1.46e−03	$1.25e-02^{***}$	$1.19e-02^{***}$	0.00e+00	0.00e+00	/	/
MCD	2017	$2.85e-02^{***}$	$3.70e-02^{***}$	$9.68e-02^{***}$	1.03e−02	0.00e+00	/	/
	2018	$8.15e-03^{***}$	$9.12e-03^{***}$	6.63e−03	0.00e+00	0.00e+00	0.00e+00	/
	2019	$1.81e-03^{**}$	$6.88e-03^{***}$	5.37e−03	0.00e+00	0.00e+00	/	/
NVDA	2017	$4.80e-03^{***}$	$2.14e-03^{**}$	0.00e+00	/	/	/	/
	2018	$4.84e-03^{***}$	1.42e−03	7.53e−03	0.00e+00	/	/	/
	2019	$3.98e-03^{***}$	$-3.50e-03^{***}$	0.00e+00	/	/	/	/
AAPL	2017	$1.36e-01^{***}$	$1.84e-01^{***}$	$3.21e-01^{***}$	$9.54e-02^{***}$	0.00e+00	/	/
	2018	$7.56e-03^{***}$	$1.17e-02^{***}$	/	/	/	/	/
	2019	$6.38e-02^{***}$	$6.84e-02^{***}$	$1.67e-01^{***}$	0.00e+00	/	/	/
ABBV	2017	$7.07e-02^{***}$	$7.59e-02^{***}$	$1.81e-01^{***}$	$3.53e-01^{***}$	$4.59e-01^{***}$	0.00e+00	/
	2018	$9.28e-03^{***}$	$1.38e-02^{***}$	7.35e−03	0.00e+00	0.00e+00	/	/
	2019	$1.50e-02^{***}$	$2.29e-02^{***}$	$4.74e-02^{***}$	/	/	/	/
PM	2017	$1.94e-02^{***}$	$1.88e-02^{***}$	$3.29e-02^{***}$	0.00e+00	0.00e+00	/	/
	2018	$3.15e-03^{***}$	$2.49e-03^{***}$	3.40e−03	0.00e+00	0.00e+00	0.00e+00	/
	2019	1.32e−03	8.93e−04	$7.86e-02^{***}$	0.00e+00	/	/	/
BAC	2017	$4.48e-01^{***}$	$4.48e-01^{***}$	$4.66e-01^{***}$	$5.60e-01^{***}$	$6.74e-01^{***}$	$8.04e-01^{***}$	$8.83e-01^{***}$
	2018	$3.87e-01^{***}$	$3.87e-01^{***}$	$3.91e-01^{***}$	$4.27e-01^{***}$	$4.64e-01^{***}$	$5.10e-01^{***}$	$5.99e-01^{***}$
	2019	$2.62e-01^{***}$	$2.63e-01^{***}$	$2.69e-01^{***}$	$2.90e-01^{***}$	$3.27e-01^{***}$	$3.90e-01^{***}$	$5.10e-01^{***}$
CSCO	2017	$3.30e-01^{***}$	$3.30e-01^{***}$	$3.33e-01^{***}$	$3.94e-01^{***}$	$4.66e-01^{***}$	$5.89e-01^{***}$	$8.06e-01^{***}$
	2018	$2.30e-01^{***}$	$2.36e-01^{***}$	$2.66e-01^{***}$	$3.05e-01^{***}$	$3.52e-01^{***}$	$4.29e-01^{***}$	$5.43e-01^{***}$
	2019	$2.78e-01^{***}$	$2.95e-01^{***}$	$3.65e-01^{***}$	$4.36e-01^{***}$	$4.68e-01^{***}$	$5.07e-01^{***}$	$2.49e-01^{***}$
KO	2017	$3.36e-01^{***}$	$3.38e-01^{***}$	$3.53e-01^{***}$	$4.00e-01^{***}$	$4.58e-01^{***}$	$5.38e-01^{***}$	$6.44e-01^{***}$
	2018	$2.25e-01^{***}$	$2.31e-01^{***}$	$2.60e-01^{***}$	$3.06e-01^{***}$	$3.56e-01^{***}$	$4.13e-01^{***}$	$4.97e-01^{***}$
	2019	$2.24e-01^{***}$	$2.29e-01^{***}$	$2.55e-01^{***}$	$3.02e-01^{***}$	$3.61e-01^{***}$	$4.46e-01^{***}$	$4.91e-01^{***}$
ORCL	2017	$3.39e-01^{***}$	$3.49e-01^{***}$	$4.03e-01^{***}$	$4.75e-01^{***}$	$5.44e-01^{***}$	$6.21e-01^{***}$	$6.97e-01^{***}$
	2018	$1.73e-01^{***}$	$1.76e-01^{***}$	$1.92e-01^{***}$	$2.27e-01^{***}$	$2.85e-01^{***}$	$3.66e-01^{***}$	$8.05e-02^{***}$
	2019	$2.38e-01^{***}$	$2.48e-01^{***}$	$3.01e-01^{***}$	$3.55e-01^{***}$	$3.94e-01^{***}$	$4.86e-01^{***}$	/
PFE	2017	$3.24e-01^{***}$	$3.27e-01^{***}$	$3.55e-01^{***}$	$4.19e-01^{***}$	$4.97e-01^{***}$	$6.17e-01^{***}$	$7.76e-01^{***}$
	2018	$1.79e-01^{***}$	$1.82e-01^{***}$	$2.07e-01^{***}$	$2.51e-01^{***}$	$2.90e-01^{***}$	$2.69e-01^{***}$	$2.09e-01^{***}$
	2019	$3.32e-01^{***}$	$3.39e-01^{***}$	$3.77e-01^{***}$	$4.47e-01^{***}$	$5.21e-01^{***}$	$5.55e-01^{***}$	$6.62e-01^{***}$
VZ	2017	$7.78e-02^{***}$	$7.80e-02^{***}$	$8.10e-02^{***}$	$1.06e-01^{***}$	$1.47e-01^{***}$	$1.82e-01^{***}$	$9.13e-02^{***}$
	2018	$1.03e-01^{***}$	$1.05e-01^{***}$	$1.14e-01^{***}$	$1.23e-01^{***}$	$1.79e-01^{***}$	$3.51e-01^{***}$	$3.54e-01^{***}$
	2019	$1.87e-01^{***}$	$1.91e-01^{***}$	$2.17e-01^{***}$	$2.61e-01^{***}$	$3.13e-01^{***}$	$3.77e-01^{***}$	$5.30e-01^{***}$
